# Loss of BOK increases vulnerability of p53 deficient non-small cell lung cancer cells to ATR inhibition through its role in uridine metabolism

**DOI:** 10.1038/s41418-026-01666-0

**Published:** 2026-01-29

**Authors:** Philippe JeanRichard, Aparna Ananthanarayan, Liyang Wu, Ali Jazaeri Jouneghani, Daniel Bachmann, Thomas Kaufmann

**Affiliations:** Institute of Pharmacology, University of Bern, Inselspital, INO-F, CH-3010 Bern, Switzerland

**Keywords:** Non-small-cell lung cancer, Cancer metabolism

## Abstract

BOK is a pro-apoptotic member of the BCL-2 family frequently repressed in cancer and with emerging roles beyond apoptosis. BOK interacts with and increases uridine monophosphate synthetase (UMPS) activity, thereby promoting uridine monophosphate (UMP) synthesis. We previously showed that BOK protein is downregulated in primary human lung cancer samples, correlating with poorer patient survival. Here, we demonstrate that BOK deficiency increases DNA damage, triggering p53 activation and cell cycle arrest in two independent non-small cell lung cancer (NSCLC) cell models that express either WT or defective p53. In a p53-deficient setting, BOK loss caused elevated baseline DNA damage rendering cells more dependent on alternative DNA repair pathways. We exploited this vulnerability by inhibiting the ATR-mediated DNA damage response pathway with the selective ATR inhibitor ceralasertib (AZD6738). ATR inhibition in BOK/p53 compound-deficient NSCLC cells exacerbated DNA damage and induced cell death, indicating a synthetic lethal interaction. The DNA damage in BOK-deficient cells was rescued by a cell permeable BOK-BH3-derived peptide, confirming the mechanistic link between BOK and UMPS. Taken together, our findings reveal a vulnerability in NSCLC, where combined loss of p53 and BOK sensitises cells to ATR inhibition. This synthetic interaction suggests that p53-deficient tumours with reduced BOK expression may be more reliant on ATR-mediated DNA repair, providing a mechanistic basis for their susceptibility to ATR inhibitors. Given the frequent inactivation of p53 in lung cancer, our study offers a rationale for clinical exploration of ATR inhibitors, in combination with standard chemotherapy, in the context of reduced BOK function. Future investigations into the broader role of BOK in genomic stability and nucleotide metabolism may uncover additional therapeutic strategies for cancers with repressed BOK.

## Introduction

Tumour-related diseases are, as of today, still a leading cause of death worldwide. Hence, it is crucial to deepen our understanding of the mechanisms driving tumour emergence and progression, as well as searching for new and more efficient treatments.

The members of the BCL-2 family have emerged as important tumour regulators, especially due to their role in regulating Apoptosis [1–3]. They can be classified into three subgroups: Anti-apoptotic proteins, pro-apoptotic BH3-only proteins and proapoptotic effector proteins [4]. During malignant transformation, many cancers find a way to avoid apoptosis through dysregulation of BCL-2 protein expression; either by upregulation of anti-apoptotic members, or by downregulation of pro-apoptotic BH3-only members [4]. The best-characterised members of the pro-apoptotic effector subfamily are BAX and BAK. During apoptosis, they become activated and oligomerize to form a pore in the mitochondrial outer membrane [2, 4, 5]. This leads to a process termed mitochondrial outer membrane permeabilization (MOMP) during which apoptogenic proteins such as cytochrome c or Smac/DIABLO are released into the cytoplasm, and which is generally considered the point of no return during apoptosis [2, 4–6].

BCL-2 related ovarian killer (BOK) was discovered in 1997 and is classified together with the effector proteins BAX and BAK, due to sequence homology and structural similarities [7]. In contrast to BAX and BAK, however, BOK was shown to be mainly localised at the endoplasmic reticulum where it was shown to interact with inositol 1,4,5-triphosphate receptor (IP3R) and to modulate ER related function, possibly at mitochondrial contact sites [8–10]. It was also shown that BOK was rather ubiquitously expressed and not, as initially assumed, in ovaries only [11]. Intriguingly, BOK was shown to be frequently deleted genomically or repressed by other means in human cancers [12] (and reviewed in Naim et al. [13]). For example, we previously showed in biopsies from non-small cell lung cancer (NSCLC) patients that BOK protein levels were downregulated in more advanced tumours compared with low grade tumours or control tissue, with high BOK levels associating with increased patient survival, thereby supporting a tumour-suppressor like function for BOK [14]. In the same study we provided evidence for epigenetic downregulation of BOK in both lung adenocarcinoma (LUAD) and lung squamous cell carcinoma (LUSC) cell lines [14].

Recently, a novel “non-apoptotic” role of BOK in the regulation of uridine metabolism was identified [15]. Specifically, BOK was shown to bind and activate the enzymatic activity of uridine monophosphate synthetase (UMPS) about threefold [15]. UMPS is a bifunctional enzyme localised in the cytoplasm, which catalyses the two final steps in the de novo synthesis of uridine monophosphate (UMP) from orotate and which is also primarily responsible for the conversion of the chemodrug 5-fluorouracil into its bioactive metabolites [16, 17]. Of note, UMPS is one of the rate-limiting enzymes in the de novo synthesis of pyrimidine nucleotides. Accordingly, BOK is important for proper uridine metabolism [15]. In cancer, dysregulation of nucleotide metabolism is known to promote malignant transformation [18, 19].

The transcription factor p53 is activated in response to multiple cellular stress signals and is one of the most important players in cancer suppression [20, 21]. In response to DNA damage p53 regulates DNA repair and causes cell cycle arrest at the G1/S-checkpoint of the cell cycle, primarily via the activation of p21. Alternatively, p53 can initiate apoptosis via transcriptional induction of the BH3-only gene PUMA [22]. In the absence of a stressor, p53 activity is kept low by the E3 ubiquitin ligase MDM2, preventing it from assembling into its active, tetrameric form [20, 21]. TP53 is frequently inactivated in NSCLC with mutations found in 50–65% of cases [23, 24]. The prevalence is highest in squamous cell carcinoma (>80%), while ca. 50% of p53 mutations are found in lung adenocarcinoma [24, 25]. Most mutations affect the DNA-binding domain, leading to loss of transcriptional activity and often stabilisation of dysfunctional p53 protein, while nonsense or frameshift mutations can abolish p53 expression [26].

Somewhat counterintuitive for a pro-apoptotic protein repressed in cancer, loss of BOK was shown to impair cell proliferation in several non-transformed as well as malignantly transformed cell types and tissues [14, 27]. We previously reported that several genes associated with cell cycle arrest were significantly upregulated upon BOK knockout, among them the p53 effector protein p21 [15, 27]. Further, we showed that the growth defect observed upon BOK knockout was dependent on p21 and p53 [27]. In a mutant KRAS^G12D^ driven mouse model of lung cancer, Meinhart et al. showed that loss of BOK results in reduced proliferation of tumour lesions and overall fewer and smaller tumours compared with controls [28]. Of note, this phenotype was completely lost on a *p53*^*-/-*^ background, further substantiating the connection of loss of BOK to p53 activation [28].

Why exactly loss of BOK results in baseline p53 activation is still unclear. It can be hypothesised that in BOK deficient cells, reduced de novo synthesis of UMP causes a lack of precursor molecules needed for pyrimidine synthesis. This would lead to a disbalance between cellular purine and pyrimidine pools, which has been shown to cause low grade DNA damage, and subsequently ATR-CHK1 dependent p53 activation [29, 30].

Ataxia telangiectasia mutated (ATM) and Ataxia telangiectasia mutated and rad3-related (ATR) are two key enzymes in DNA damage response pathways that a cell can employ in response to DNA stress [29, 31–34]. Whereas ATM is activated by double strand DNA breaks, ATR is activated by replication protein A (RPA) coated single strand DNA breaks, which occur on stalled replication forks or during repair by homologous recombination [32]. ATR is recruited to RPA coated single stranded DNA by a protein called ATRIP (ATR interacting protein), forming a complex together with an additional protein called TOPB1. In this complex, ATR becomes fully active and can phosphorylate its downstream target CHK1, initiating a cascade, which will eventually lead to cell cycle arrest at the G2/M-checkpoint of the cell cycle, mediated by the serine-threonine kinase CDK1 [32, 35]. There is also crosstalk between CHK1 and p53, which allows the ATR pathway to also indirectly activate p53 substrates [29, 32]. p53 on the other hand, has been shown to be able to also induce G2 cell cycle arrest via its target p21 interacting with CDK1 [29, 31]. DNA damage response pathways have been subject of intense study and a lot of effort has gone into targeting it for cancer therapy. Several inhibitors of DNA damage response have been developed and are clinically studied [31–33, 36–38]. Among these, ceralasertib (AZD6738) is a specific inhibitor of the ATR enzyme [31, 36, 37]. Multiple clinical trials with ATR inhibitors are currently being conducted in cancer, including lung cancer, as monotherapies or as part of combinatorial regimens with other chemodrugs (reviewed in [39]).

Here, we demonstrate that BOK deficiency leads to a p53 dependent cell cycle arrest in NSCLC cells. Of note, upon loss of functional p53, BOK deficient cells show increased levels of DNA damage. We demonstrate using the specific ATR inhibitor ceralasertib that the phenotype of Bok^-/-^p53^-/-^ cancer cells can be exploited by inhibiting ATR-mediated cell cycle arrest and DNA repair, resulting in exacerbated DNA damage and cell death compared with p53-deficient cells. We therefore propose a model in which the loss of BOK in a p53 deficient setting leads to a state of chronic DNA-stress. A state which could explain the relatively frequent downregulation of BOK observed in human cancers. Additionally, we show that this state can be exploited by ATR inhibition with ceralasertib.

## Results

### BOK deficiency alters nucleotide metabolism and engages p53-mediated proliferation control

**Fig. 1:**
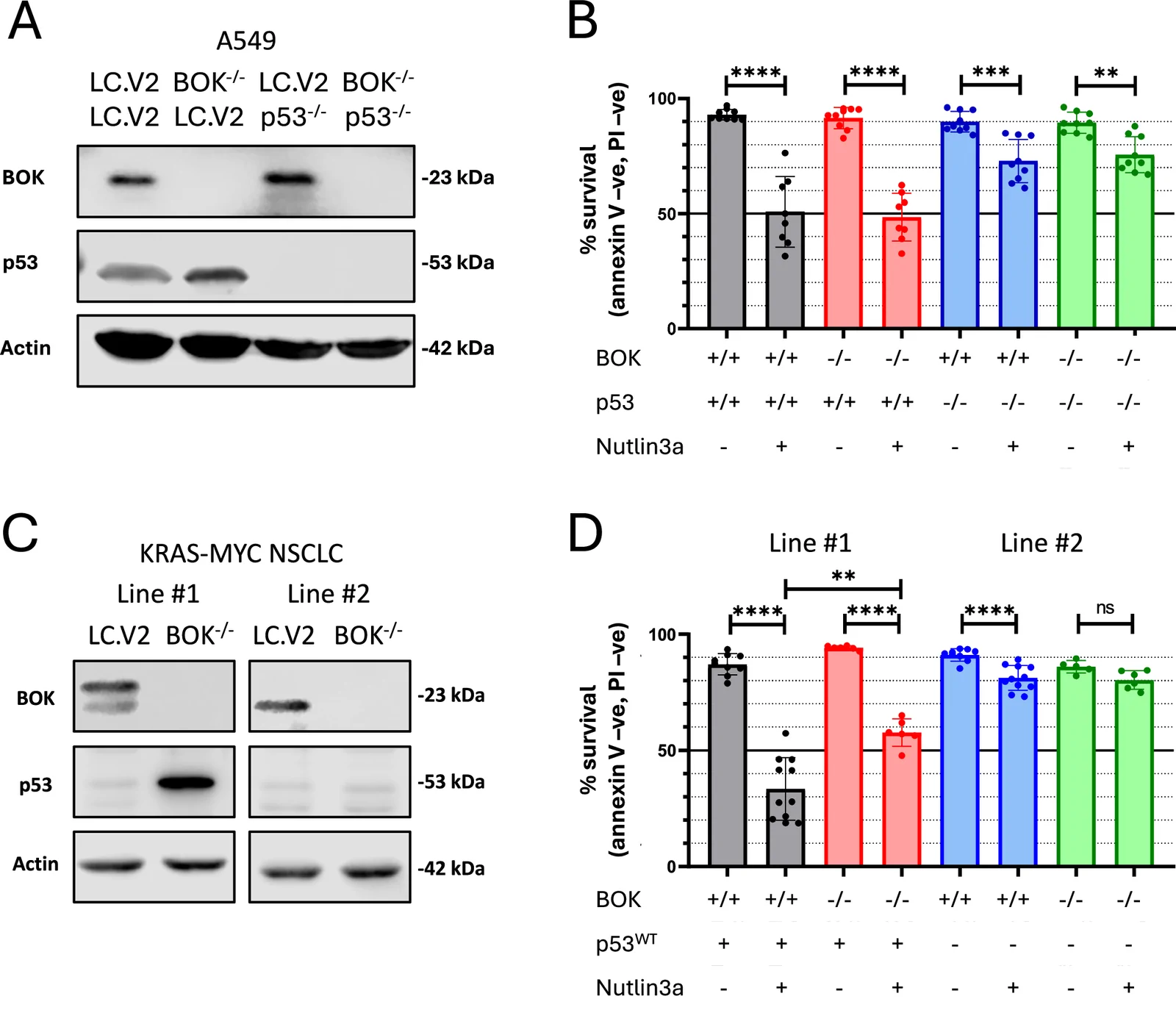
Cell model characterisation. A549 lung cancer cells, originally WT for p53, with or without CRISPR induced BOK and/or p53 deletion. **A** Western blot analysis demonstrating efficient loss of BOK and p53 expression. **B** Viability data by flow cytometry (AnnexinV/PI exclusion) of cells treated with 20 µM Nutlin 3a for 48 h (*N* = 9). Murine cell lines derived from mice, originally on a WT p53 genetic background, with mutant KRAS-MYC driven NSCLC, with or without a CRISPR induced deletion of BOK. Whereas line #1 still expresses WT p53, line #2 has lost functional p53 due to loss-of-function missense mutations **C** Western blot demonstrating BOK knockout efficacy and induction of p53 in line #1 but not line #2. **D** Viability data by flow cytometry (AnnexinV/PI exclusion) of cells treated with 20 µM Nutlin 3a for 48 h (*N* = 9). Statistical analysis was performed by two-way ANOVA, followed by pairwise Bonferroni-adjusted T-tests. *: *p* ≤ 0.05; **: *p* ≤ 0.01; ***: *p* ≤ 0.001; ****: *p* ≤ 0.0001.

To investigate the effects of BOK deficiency in a p53 dependent context in lung cancer, we generated two independent cell models. As a first model, we used the well- established and p53 wildtype human lung adenocarcinoma cell line A549, in which we deleted *BOK* and/or *TP53* by CRISPR/Cas9 mediated gene editing (Fig. 1A). To prove loss of function of p53, we treated the respective lines with the MDM2 inhibitor Nutlin 3a, thereby inducing p53-dependent cell death [22]. As shown in Fig. 1B, Nutlin 3a induced significant cell death in p53^+/+^ cells, but much less so in p53^-/-^ cells, proving functional loss of p53. As a second model, we used cell lines derived from tumours from a mouse NSCLC model driven by mutant KRAS^G12D^ with additional moderate but constitutive overexpression of c-MYC. These mice were originally on a p53 wildtype genetic background [40]. We first wanted to test the p53 status of these cell lines, using western blotting and viability assays after Nutlin 3a treatment. As shown in Fig. 1C and D, we obtained cell lines with either retained functional p53 or with loss-of-function p53 mutation. Sequencing of the TP53 gene in this cell line confirmed the presence of missense loss-of-function mutations in the DNA-binding region among others (see Supplementary Table S1). We then deleted Bok in these lines by CRIPSR/Cas9 mediated gene editing to investigate if the phenotypes observed in Bok^-/-^ cells, especially the proliferation defect, were still present in the context of c-MYC overexpression. In primary experiments with these cell lines, the BOK deletion in p53^WT^ cell line #1 was gradually lost over time, which was likely due to a selection disadvantage caused by the proliferation defect in BOK-deficient cells (see below). To alleviate this issue, we used a pool of confirmed BOK-deficient subclones for this line.

To additionally verify the validity of our models, we confirmed our previously reported interaction between BOK and UMPS at the endogenous level by co-immunoprecipitation assays for both cell models (Supplementary Fig. S4) [15].

**Fig. 2:**
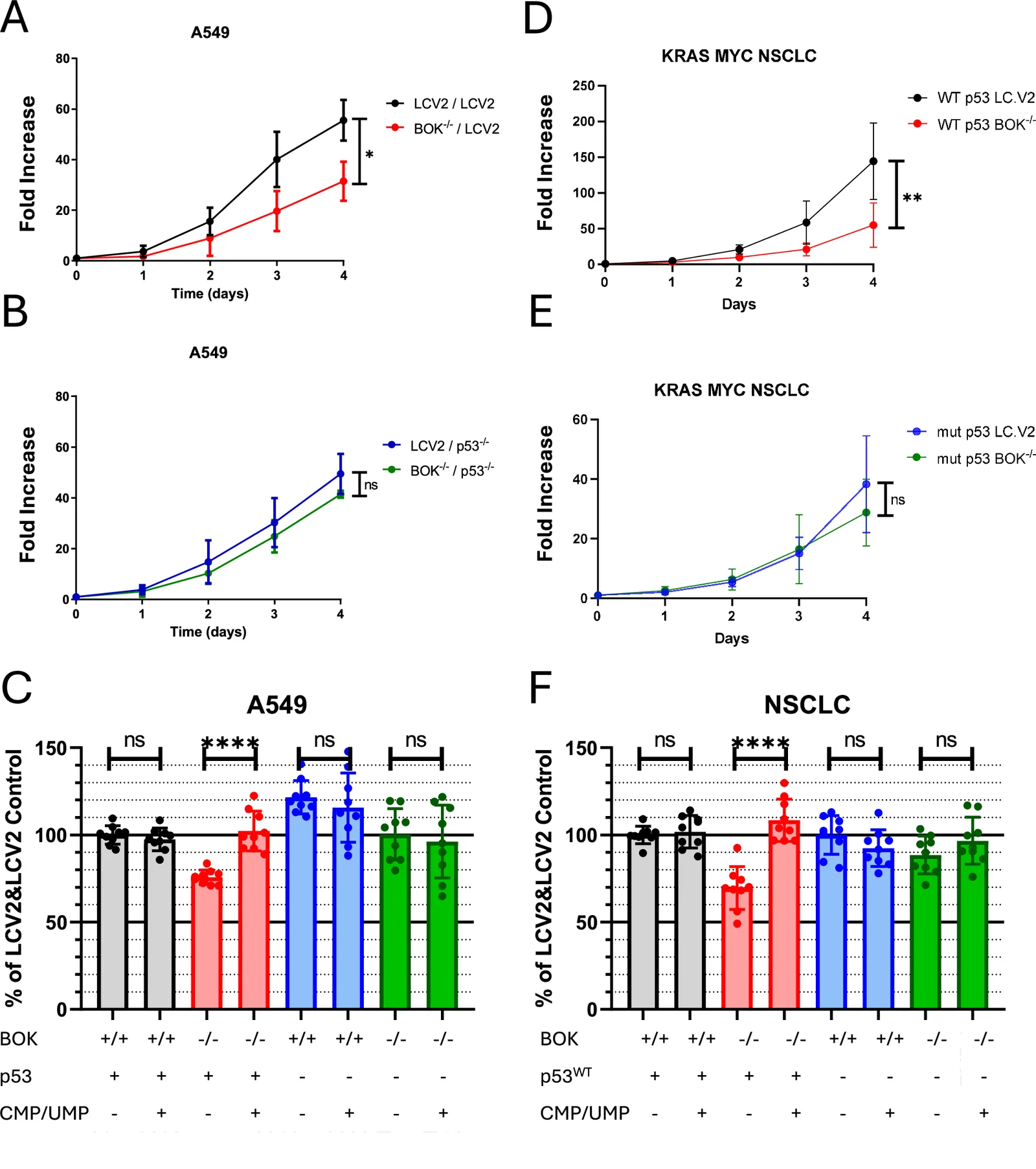
BOK-deletion results in decreased proliferation in a p53 dependent manner. Viable cell numbers were measured over a period of four days using the PrestoBlue metabolic assay comparing BOK-proficient vs. BOK deficient cells. **A** A549 cells with a functional p53, *N* = 4. **B** p53-deficient A549 cells, *N* = 4. **C** A549 cells were left to grow for a period of 72 h, with or without supplementation with 100 µM CMP and 1 mM UMP. **D** KRAS-MYC NSCLC cells with a functional p53, *N* = 4. **E** KRAS-MYC NSCLC cells with a mutated p53, *N* = 4. **F** KRAS-MYC NSCLC cells were left to grow for a period of 72 h, with or without supplementation with 100 µM CMP and 1 mM UMP. Statistical analysis was performed by two-way ANOVA, followed by pairwise T-tests Bonferroni-adjusted (C, F) or by T-tests (A, B, D, E). *: *p* ≤ 0.05; **: *p* ≤ 0.01; ***: *p* ≤ 0.001; ****: *p* ≤ 0.0001.

In order to investigate whether loss of BOK results in p53 dependent proliferation defects in our cell models, cellular proliferation was assessed over time. The results showed that loss of BOK in A549 cells resulted in a significant decrease in proliferation and that this effect was completely lost when p53 was additionally lost (Fig. 2 A, B). Importantly, a significant decrease in proliferation of BOK-deficient cells was also observed in the murine KRAS-MYC cells, which again was entirely p53 dependent (Fig. 2D, E). These data indicate that constitutive c-MYC expression could not override p53-induced cell cycle arrest in that cellular context. Based on our hypothesis, we expected that these p53 dependent growth defects are caused by the lack of UMPS activity. This would lead to a lack of UMP for pyrimidine synthesis. To test this, we supplemented the cell medium with UMP and with CMP. The latter is needed in addition to UMP, as CTP is synthesised from UMP via UTP, with the conversion of UTP to CTP by CTP synthase representing the rate-limiting step [41–44]. Supplementation of BOK^-/-^ p53^WT^ cells with UMP and CMP restored growth rates to levels comparable with BOK proficient cells in both cell models, whereas the same supplementation did not affect growth in BOK^-/-^ cells carrying non-functional p53 (Fig. 2C, F).

### BOK/p53 compound deficiency increases susceptibility of NSCLC cells to ATR-inhibition-induced DNA damage

**Fig. 3:**
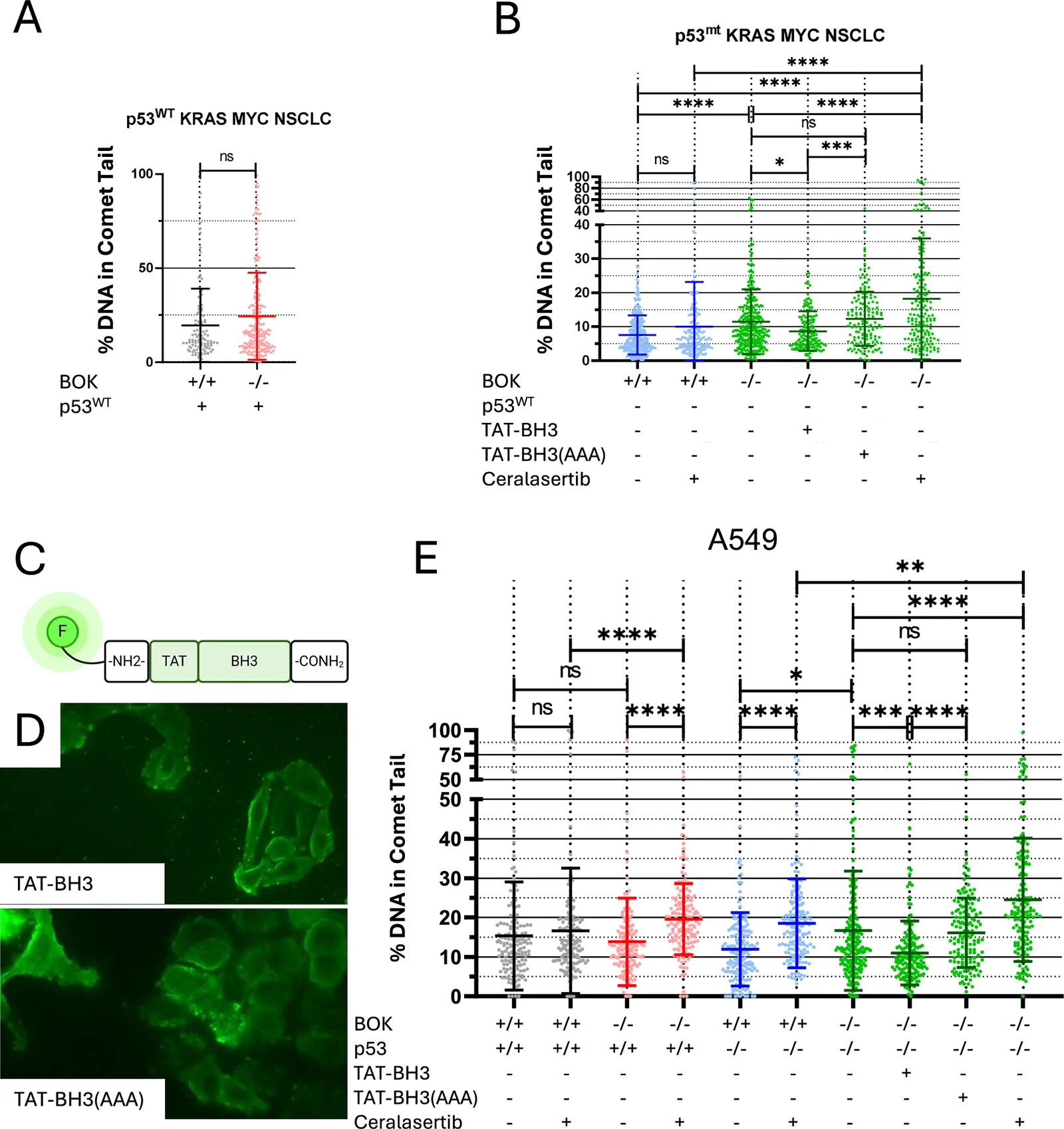
DNA damage quantification by comet assay. DNA damage at the level of individual cells was quantified using the comet assay. For each condition at least 100 nuclei from at least 3 independent experiments were quantified. Results are displayed as percent of signal in the comet tail. **A** KRAS-MYC NSCLC cells with a functional p53 (*N* ≥ 100). **B** KRAS-MYC NSCLC cells with a mutated p53 (*N* ≥ 150). Cells in **A** and **B** were treated with 10 µM of a cell-permeable TAT-fused peptide containing a region spanning the WT or mutated BH3 domain of BOK (TAT-BH3 or TAT-BH3(AAA)) for 24 h, or with 0.5 µM ceralasertib for 72 h. **C** Schematic representation of the TAT-peptide construct. A FITC-fluorophore was coupled N-terminally to the cell penetrating HIV-TAT sequence and a peptide of interest. **D** Confocal microscopy images of A549 cells loaded with 10 µM of the TAT-BH3 (top) or of the TAT-BH3(AAA) (bottom) peptides for 30 min. **E** A549 cells of the indicated genotypes were treated with 10 µM of TAT-BH3 or TAT-BH3(AAA) peptide for 24 h, or with 1.5 µM ceralasertib for 72 h prior to analysis by comet assay (*N* = 150). Statistical analysis was performed by Kruskal-Wallis test, followed by pairwise Bonferroni-adjusted Mann-Whitney tests (B + E) or by Mann-Whitney test (A). *: *p* ≤ 0.05; **: *p* ≤ 0.01; ***: *p* ≤ 0.001; ****: *p* ≤ 0.0001.

Based on these results and previous published work [15, 28], we hypothesised that in our BOK-deficient cells, low grade DNA damage would occur at baseline. To investigate this, comet assays were performed. We found that in both of our cell models, loss of BOK does not result in a significant increase of DNA damage when p53 was functional; however, such an increase became obvious and significant in both A549 and KRAS-MYC models upon additional loss of p53 (Fig. 3A, B, E). BOK^+/+^p53^mt^ KRAS-MYC cells (line #2) displayed an average of 7.56% (SD = 5.75%) signal in the comet tail, compared with 11.45% (SD = 9.57%) upon loss of BOK (*p* < 0.0001) (Fig. 3B). A549 p53^-/-^ cells displayed 11.95% (SD = 9.31%) signal compared with 16.68% (SD = 15.18%) in *BOK*^*-/-*^*p53*^*-/-*^ cells (p = 0.0237) (Fig. 3E). We then performed a rescue experiment using a fluorescent, cell-penetrating HIV1 TAT-based fusion peptide spanning either the wildtype BOK BH3 domain, or a mutant form termed BH3(AAA), which was previously shown to fail to interact with UMPS and to fail to rescue the proliferation defect seen in BOK-null cells (Fig. 3C + Supplementary Fig. S1–3) [15, 27]. As shown in Fig. 3B and E, preloading of BOK/p53 compound-deficient A549 or p53^mt^ KRAS-MYC NSCLC cells with FITC-TAT-BOK-BH3 peptide, but not with FITC-TAT-BOK-BH3(AAA) peptide, reduced the levels of DNA damage to levels seen in the respective BOK-proficient controls. For BOK/p53 compound-deficient A549 cells the BOK-BH3 peptide caused average tail signal to drop to 11.03% (SD = 8.11%) (*p* < 0.0001 vs. to untreated cells), whereas cells treated with the BOK-BH3(AAA) peptide retained a signal of 16.15% (SD = 8.85%) (ns vs. untreated cells). For BOK^-/-^p53^mt^ KRAS-MYC NSCLC cells the BOK-BH3 peptide reduced tail signal to 8.68% (SD = 5.83) (*p* = 0.0451 vs. untreated cells), while the BOK-BH3(AAA) peptide resulted in an average signal of 12.32% (SD = 7.98%) (ns vs. untreated cells). Taken together, these data demonstrate that DNA damage in BOK-deficient cells is increased upon loss of p53, and that this phenotype depends on the BH3 domain of BOK, which has been shown to be critical for the interaction with UMPS.

These findings prompted us to sensitise BOK/p53 compound-deficient cells to DNA damage by inhibiting the ATR pathway, a key mediator of DNA repair and genome stability [35]. To investigate this, the specific pharmacological ATR inhibitor ceralasertib was used (IC_50_ of ca. 1 nM against the isolated enzyme) [32, 37]. Ceralasertib treatment did not cause a significant increase in DNA damage in parental A549 cells; with the average tail signal remaining at 16.66% (SD = 15.94%) versus 15.33% (SD = 13.72%) (ns) in untreated cells (Fig. 3E). However, upon loss of either BOK or p53, DNA damage was significantly increased: in BOK^-/-^ cells to 19.59% (SD = 9.05%) versus 13.86% (SD = 11.14%) in untreated cells (*p* < 0.0001) and in p53^-/-^ cells to 18.55% (SD = 11.34%) versus 11.95% (SD = 9.314%) in untreated cells (*p* < 0.0001) (Fig. 3E). Importantly, this increase was becoming much more prominent upon combined loss of BOK and p53, where the average signal increased to 24.53% (SD = 15,67%) versus 16.68% (SD = 15.18%) in untreated cells (*p* < 0.0001) (Fig. 3E). A similar response was seen in p53^mt^ KRAS-MYC NSCLC cells, which were most sensitive to ceralasertib treatment when BOK was additionally deleted (18.22%, SD 17.80%; *p* < 0.0001 BOK^+/+^ vs. Bok^-/-^) (Fig. 3B). Taken together, these results point towards an increased susceptibility of p53-deficient lung cancer cells that have additionally lost BOK, compared with either p53- or BOK single deficient cells.

**Fig. 4:**
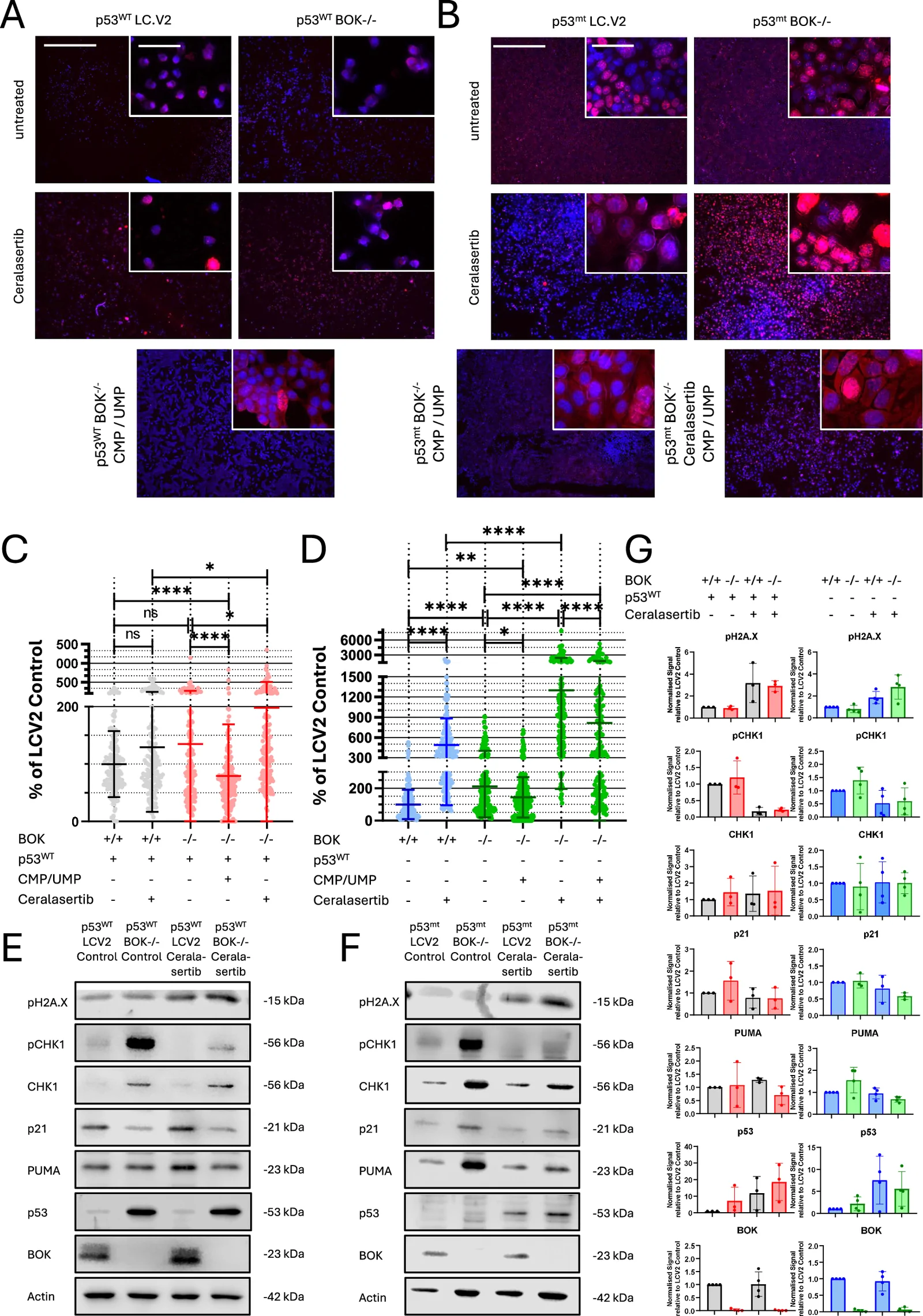
DNA damage quantification by pH2A.X staining in KRAS-MYC NSCLC cells. **A–D** pH2A.X signal per cell quantification by fluorescence microscopy. The scale bars in representative images represent 1 mm and 50 µm, respectively. The results are presented as percentage of the average content in control (empty LCV2) cells. Cells were treated with 0.5 µM ceralasertib and/or with 100 µM CMP and 1 mM UMP as indicated for 72 h. **A**, **C** WT p53 cells with or without BOK. **B**, **D** Mutant p53 cells with or without BOK. (*N* = 150 in **C,** **D**). pH2A.X measurement by western blotting. Cells were treated with 0.5 µM ceralasertib for 72 h. **E** WT p53 cells with or without BOK. **F** Mutant p53 cells with or without BOK. **G** Quantification of western blot signals from **E**, **F**. Signals were normalised to β-actin and plotted as fold increase relative to LCV2 controls (*N* = 3). Statistical analysis was performed by Kruskal-Wallis test, followed by pairwise Bonferroni-adjusted Mann-Whitney tests. *: *p* ≤ 0.05; **: *p* ≤ 0.01; ***: *p* ≤ 0.001; ****: *p* ≤ 0.0001.

To further substantiate the effects seen on the DNA damage response, pH2A.X (Ser139) signal was quantified by immunofluorescence microscopy in both cell models. At baseline, significant increase in H2A.X phosphorylation was seen in BOK p53^mt^ KRAS-MYC NSCLC cells with the average signal at 211.4% (SD = 191.5%) of empty vector control (*p* < 0.0001) (Fig. 4B, D and Suppl. Fig. S6). In p53^WT^ cells by comparison, baseline levels of H2A.X phosphorylation were only marginally elevated to 134.9% (SD = 134.3%) upon BOK loss (ns) (Fig. 4A, C). The baseline increase in H2A.X phosphorylation upon loss of BOK could be rescued by UMP + CMP supplementation in both p53 proficient (79.23% (SD = 89.98%)) (*p* < 0.0001 vs. untreated cells) and p53 deficient (151.7% (SD = 127.0%)) (*p* = 0.0123 vs. untreated cells) cells (Fig. 4A–D). Upon ceralasertib treatment a significant increase in DNA damage was seen in all genotypes, regardless of the BOK and p53 status; however, the increase was again more pronounced in BOK deficient cells and significantly exacerbated upon additional loss of p53. Specifically, signal after ceralasertib increased to 129.4% (SD = 112.5%) in BOK^+/+^p53^WT^ (ns vs. untreated), to 198.1% (SD = 306.3%) in BOK^-/-^ (*p* = 0.0202 vs. untreated), to 488.7% (SD = 393.4%) in p53^mt^ (*p* < 0.0001 vs. untreated) and to 1301% (SD = 1105%) in BOK^-/-^p53^mt^ cells (*p* < 0.0001 vs. untreated). Furthermore, UMP + CMP supplementation protected BOK^-/-^p53^mt^ cells from the effects of ATR inhibition, reducing the signal to 816.5% (SD = 946.8%) (*p* < 0.0001 vs. ceralasertib treated) (Fig. 4B, D).

**Fig. 5:**
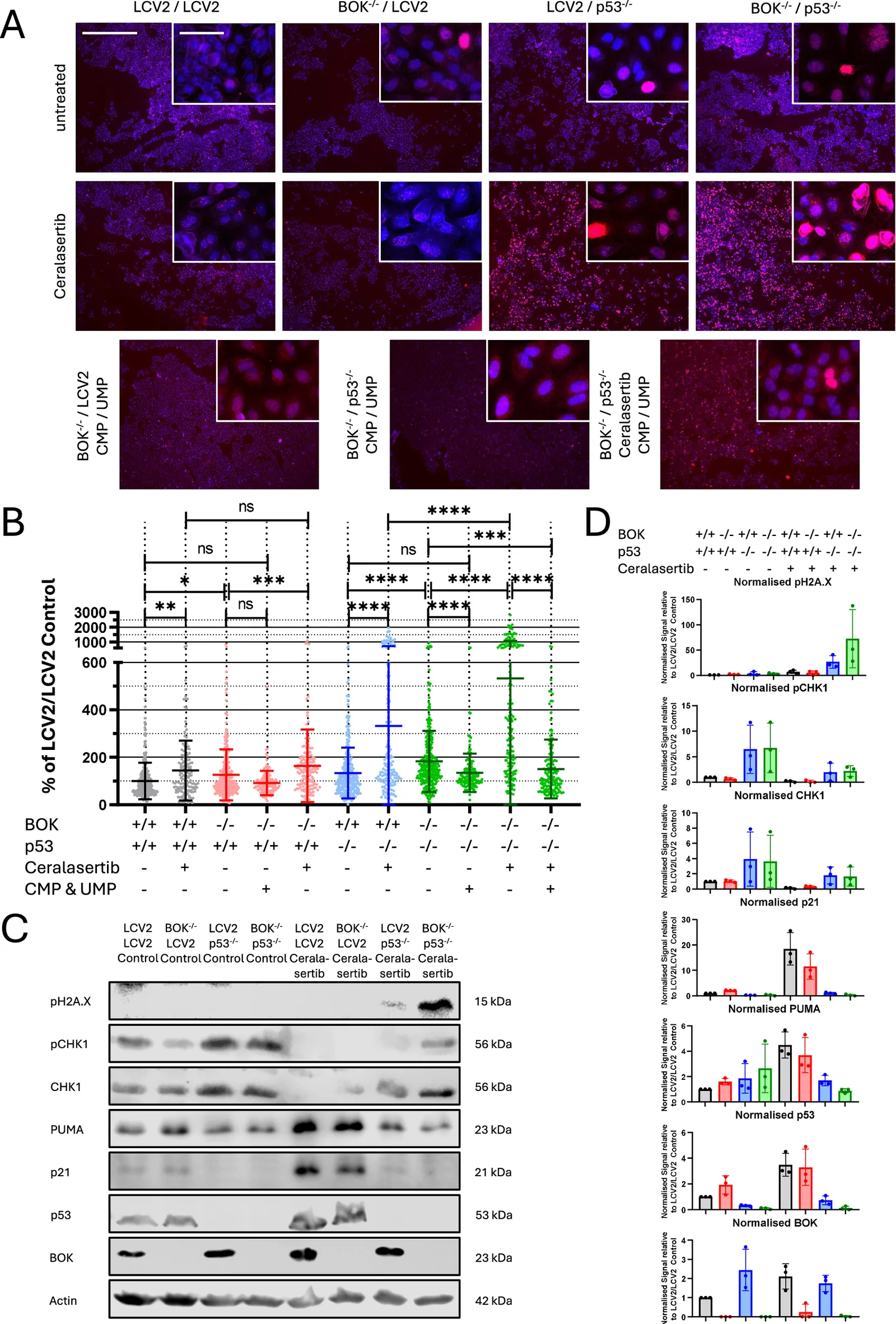
DNA damage quantification by pH2A.X staining in A549 cells. **A** Representative images of fluorescence microscopy images of A549 cells of indicated genotypes used for quantification in B. Scale bars represent 1 mm and 50 µm, respectively. **B** quantification of pH2A.X signal per cell by fluorescence microscopy. Values are presented as percentage of the average content in control (LCV2 + LCV2) cells. Cells were treated with 1.5 µM ceralasertib and/or with 100 µM CMP and 1 mM UMP as indicated for 72 h. *N* = 150 **C** Western blot analysis of p-H2A.X. Cells were treated with 1.5 µM ceralasertib for 72 h. **D** Quantification of western blot signals from **C**. Signals were normalised to β-actin and plotted as fold increase relative to LCV2/LCV2 control signal, *N* = 3. Statistical analysis was performed by Kruskal-Wallis test, followed by pairwise Bonferroni-adjusted Mann-Whitney tests. *: *p* ≤ 0.05; **: *p* ≤ 0.01; ***: *p* ≤ 0.001; ****: *p* ≤ 0.0001.

Similar results were obtained in A549 cells. Ceralasertib treatment only caused small – but significant - increase in H2A.X phosphorylation in p53 proficient cells, regardless of BOK status. Specifically, to 144% (SD = 126.2%) in BOK^+/+^p53^+/+^ cells (*p* = 0.0024 vs. untreated) and to 164.2% (SD = 153.3%) in BOK^-/-^ cells (*p* < 0.0001 vs. untreated) (Fig. 5A, B). However, in p53^-/-^ A549 cells, BOK deficiency alone was sufficient to cause a measurable increase in H2A.X phosphorylation, to an average of 182.6% (SD = 128.5%) compared with 133.8% (SD = 107.0%) in p53^-/-^ cells (*p* < 0.0001). This was further enhanced upon ceralasertib treatment, specifically, to 332.2% (SD = 394.5%) in p53^-/-^ cells (*p* < 0.0001 vs. untreated) and to 532.9% (SD = 545.6%) in BOK^-/-^p53^-/-^ cells (*p* < 0.0001 vs. untreated) (Fig. 5A, B). Similar to the mouse model, UMP + CMP supplementation lowered both pH2A.X levels in BOK deficient cells and provided protection from the effects of ATR inhibition (Fig. 5A, B). Specifically, levels decreased down to 91.47% (SD = 51.23%) in BOK^-/-^ cells (ns vs. untreated), to 134.9% (SD = 80.98%) (*p* < 0.0001 vs. untreated) and 150.5% (SD = 123.8%) (*p* < 0.0001 vs. ceralasertib treated) in BOK^-/-^p53^-/-^ cells (Fig. 5A, B).

Additionally, whereas pH2A.X was not detectable by western blotting in untreated or p53 proficient A549 cells, a small signal was seen in ceralasertib treated *BOK*^+/+^p53^-/-^ cells, which increased upon additional loss of BOK (Fig. 5C, D). Ceralasertib treatment of p53 proficient cells resulted in an upregulation of p21 and PUMA, suggesting that these damaged cells are either directed towards cell cycle arrest or apoptosis in a p53 dependent manner (Fig. 5C). In KRAS-MYC NSCLC cells with a mutated p53, in which ceralasertib treatment led to an increased phosphorylation of H2A.X, which was slightly but not significantly increased upon loss of BOK. In KRAS-MYC NSCLC with a functional p53, ceralasertib also lead to detectable changes in H2A.X phosphorylation, which were however not affected by loss of BOK (Fig. 4E–G). Additionally, in all cell models ceralasertib treatment led to a significant reduction in phosphorylation of the ATR target CHK1, confirming the inhibition of ATR (Fig. 4E–G, Fig. 5C, D).

### BOK/p53 compound deficiency creates synthetic lethality upon ATR inhibition in NSCLC cells

**Fig. 6:**
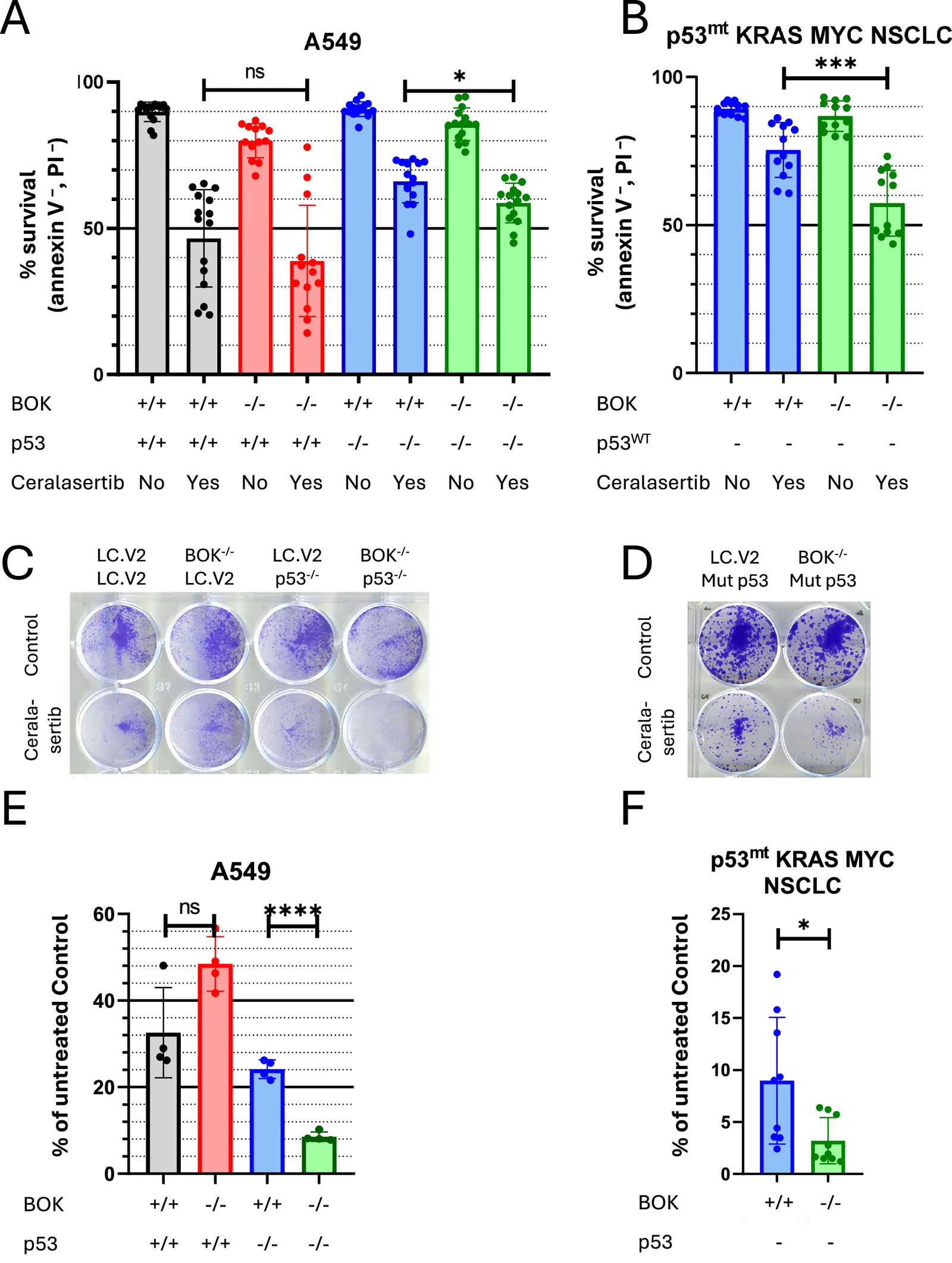
Cell survival after ceralasertib treatment. **A** Cell survival of A549 cells treated for 10 days with 1.5 µM ceralasertib was quantified by flow cytometry using AnnexinV/PI exclusion. Survival was defined as the percentage of AnnexinV/PI double negative cells (*N* = 12). **B** Cell survival of mutant p53 KRAS-MYC NSCLC cells treated for 6 days with 0.5 µM ceralasertib was quantified by flow cytometry using AnnexinV/PI exclusion (*N* = 12). **C**, **D** Representative images of colony formation assays using crystal violet staining. A549 cells were treated for 4 days with 1.5 µM ceralasertib; mutant p53 KRAS-MYC NSCLC cells were treated for 6 days with 0.5 µM ceralasertib. **E**, **F** Quantification of colony formation assays from **C** and **D**. Colony numbers were plotted as percentage of untreated control (E: *N* = 4, F: *N* = 9). Statistical analysis was performed by two-way ANOVA, followed by pairwise Bonferroni-adjusted T-tests. *: *p* ≤ 0.05; **: *p* ≤ 0.01; ***: *p* ≤ 0.001; ****: *p* ≤ 0.0001.

To assess whether the treatment with ceralasertib affects cancer cell survival, cell viability was assessed by flow cytometry using Atto488-AnnexinV and propidium iodide staining. Furthermore, long term survival by colony formation was assessed using crystal violet staining. The results confirmed that BOK deficient cells are slightly, but significantly more sensitive to ATR pathway inhibition in both cell models (Fig. 6A, B). In A549 cells there were on average 46.61% surviving cells (SD = 16.63%) in BOK^+/+^p53^+/+^ cells versus 38.88% (SD = 19.04%) in BOK^-/-^ (ns) and 66.11% (SD = 7.366) in p53^-/-^ cells versus 58.67% (SD = 6.779%) in BOK^-/-^p53^-/-^ cells (*p* = 0.0152) (Fig. 6A). In KRAS-MYC p53^mt^ NSCLC there were on average 75.33% surviving cells (SD = 9.247) in BOK-proficient versus 57.39% (SD = 11.13%) in BOK-deficient cell (*p* = 0.0003) (Fig. 6B). Of note, colony formation assays revealed that treatment with ceralasertib led to a strong decrease in colony numbers in both cell models specifically in BOK/p53 compound-deficient cells (Fig. 6C–F). While ceralasertib reduced the number and size of colonies in all cells, there was a marked difference in BOK deficient cells. This decrease in colony size and number was especially dramatic in A549 cells lacking both BOK and p53, in which colony numbers were on average reduced to 8.57% of untreated control (SD = 1.097%) compared with 24.15% (SD = 2.151%) in p53^-/-^ cells (*p* < 0.0001) (Fig. 6C+E). Similar results were seen in the p53^mt^ KRAS-MYC NSCLC cells lacking BOK, in which ceralasertib reduced colony numbers to an average of 3.202% (SD = 2.221%) of untreated control in BOK^-/-^ cells versus 8.977% (SD = 6.083%) in BOK^+/+^ cells (*p* = 0.0166). Taken together, these results show that cells with deficiencies in both BOK and p53 are increasingly sensitive to ATR inhibition.

## Discussion

We and others have reported that BOK may modulate tumour development and that several cancers may repress BOK expression to become more resistant to oncogenic stress or chemotherapy [12, 14, 15, 27, 28, 45]. For example, colorectal cancer cells downregulate BOK expression and gain resistance towards the chemodrug 5-fluorouracil (5-FU); in that context BOK has been proposed to be a potential biomarker for therapy outcome [15, 45]. In NSCLC, we reported that BOK protein is downregulated in more advanced cancer stages, correlating with poorer patient survival [14]. Although BOK resembles the effector proteins BAX and BAK in many ways, there is evidence to suggest that these tumour modulating effects may not be entirely linked to a classical apoptotic function of BOK, but rather to so-called non-apoptotic functions, such as modulation of ER stress response (reviewed in [13]). In fact, non-apoptotic functions have been reported for many, if not all, BCL-2 family members (reviewed in [46]).

A more recent non-apoptotic function of BOK is its interaction with UMPS in the cytoplasm, thereby affecting de novo pyrimidine synthesis and bioactivation of 5-FU [15]. This work helped to explain our previously reported findings that loss of BOK results in a proliferation defect in carcinomas in vitro and in vivo, a phenotype that was entirely p53 dependent [14, 27, 28]. Given that many carcinomas lose p53 function during their oncogenic transformation, it could be argued that repression of BOK becomes particularly beneficial to cancer cells expressing defective p53, as this will no longer impede proliferation, yet still confer resistance towards therapy.

Here we provide evidence that loss of BOK has profound effects on DNA damage accumulation and cellular proliferation, with significant implications for the role of the ATR pathway in mediating DNA damage response. Our findings demonstrate that BOK-deficient cells experience a proliferation defect, which is entirely dependent on the activation of p53. However, once p53 is lost or rendered non-functional, this proliferation defect is abolished, and a significant increase in baseline DNA damage is observed. This suggests that BOK is involved in maintaining genomic stability, likely through its role in uridine metabolism, and that its loss creates a state of chronic DNA stress. We propose that in the absence of BOK, cells are in a state of constant low-level genomic stress caused by a basal DNA damage. This would result in p53 activation and p53-dependent cell cycle arrest and DNA repair. However, in the absence of p53, these repairs cannot occur, rendering the cells in a measurable state of genomic stress and dependent on fewer repair options. This also opens the possibility for increased reliance on other, p53-independent DNA repair pathways, such as the ATR-CHK1 checkpoint pathway.

**Fig. 7:**
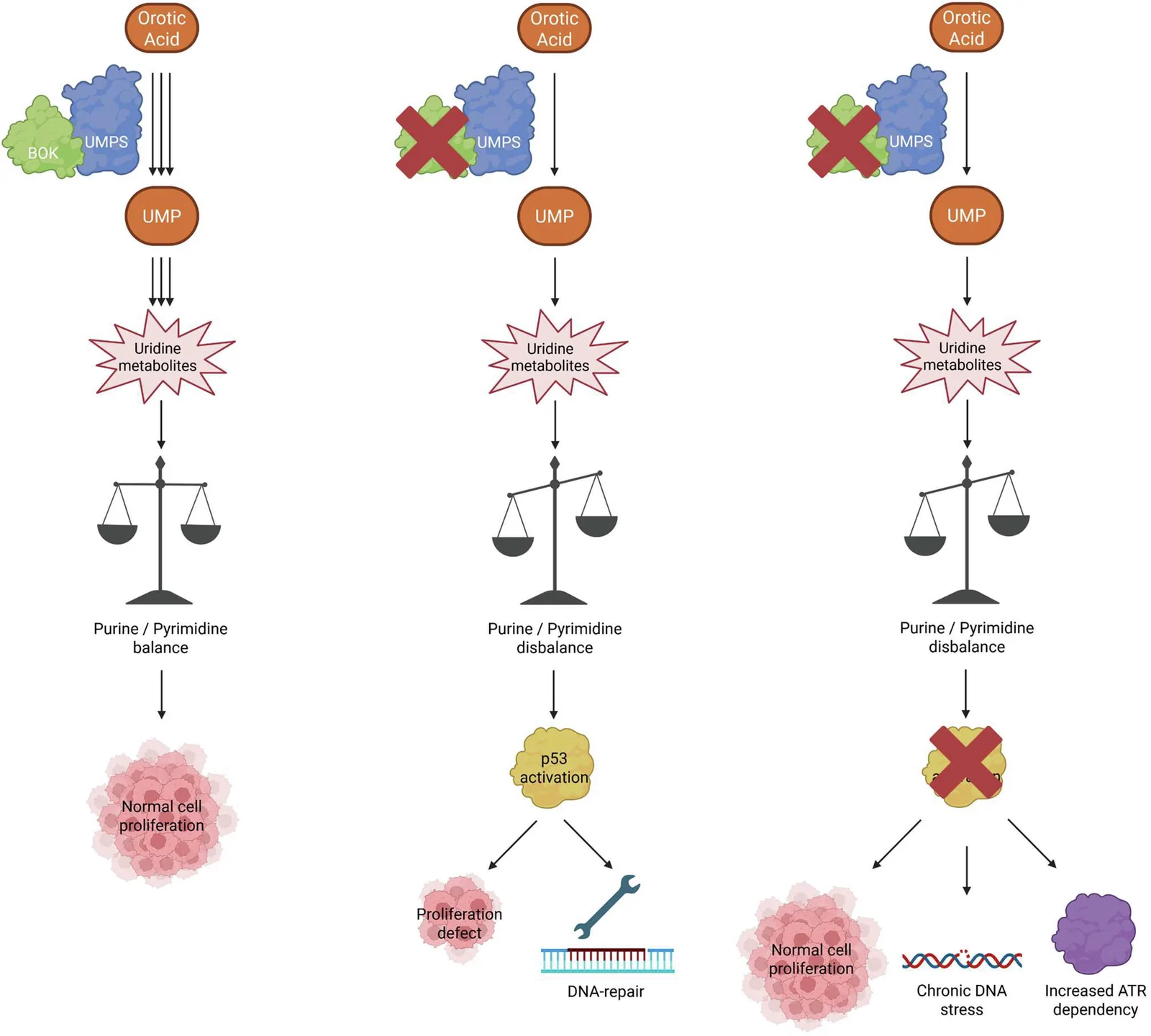
Proposed model. In the proposed model, loss of BOK reduces the catalytic activity of UMPS in the cytoplasm, resulting in an imbalance of purine and pyrimidine nucleotide pools and chronic low-grade DNA stress. This activates p53, leading to cell cycle arrest, reduced proliferation, and repair of the accumulating DNA damage. When p53 is dysfunctional, however, neither cell cycle arrest nor the associated proliferation defect occurs. Under these conditions, the cell must instead rely on a more limited set of DNA-repair pathways, such as the ATR-dependent response. Figure was created with BioRender.com.

The ATR-CHK1 pathway, which is critical for responding to replication stress, becomes increasingly important in the absence of p53, as it serves as an alternative mechanism for maintaining cell cycle arrest and genome integrity [31]. Our experiments with ATR inhibition provide direct evidence that BOK-deficient, p53-deficient/mutant cells are highly susceptible to ATR inhibition, leading to exacerbated DNA damage and reduced cell viability (Fig. 7). This effect is particularly evident with the use of ceralasertib, a selective ATR inhibitor, which increases DNA damage markers such as pH2A.X or comet assay, and results in enhanced cell death of BOK/p53 double-deficient cells compared to respective single-deficient controls for both the human (A549) and murine (KRAS-MYC NSCLC) cell models used in this study.

Notably, this phenotype could be reversed by introducing a wild-type BOK BH3 domain-containing TAT-peptide, but not a mutant BH3(AAA) peptide, underscoring the importance of BOK’s BH3 domain in this process. As the BOK-BH3(AAA) mutant was previously shown to fail to interact with UMPS, and to fail to rescue the proliferation defect [15], our findings – assuming that the TAT-BH3 peptide binds and activates UMPS in a similar way to the BOK protein - support the idea that BOK’s role in DNA repair is linked to its interaction with UMPS and the subsequent regulation of nucleotide metabolism. The connection of our observation to nucleotide metabolism was further strengthened by the rescue experiments using UMP + CMP supplementation (Figs. 2C, F, 4A–D and 5A, B). The ATR pathway, which normally compensates for such DNA stress, becomes overwhelmed when BOK and p53 are simultaneously lost, leading to a situation where even minor replication stressors result in catastrophic DNA damage.

Taken together, our study highlights a critical and previously underappreciated relationship between BOK, p53, and ATR signalling in lung cancer. The synthetic lethal interaction between BOK and p53 deficiencies suggests that BOK loss could serve as a predictive biomarker for sensitivity to ATR inhibitors, particularly in cancers with defective p53. However, whereas BOK is frequently repressed in primary NSCLC, it is rarely completely missing, and the threshold levels below which BOK must fall to result in the reported synthetic lethality remains to be determined. Nevertheless, given that p53 mutations are among the most common alterations in human cancers, our findings provide a strong rationale for further exploring ATR inhibitors as a targeted therapy for tumours with reduced BOK levels.

Future studies should aim to expand these findings in in vivo models to better understand how BOK deficiency contributes to tumour progression and therapeutic response in a whole-organism context. Additionally, investigating whether ATR inhibitor sensitivity correlates with levels of reduced but not fully deleted BOK will provide valuable insights into the clinical applicability of our findings. Importantly, it will be crucial to test combinatorial approaches of ATR inhibitors with standard-of-care chemotherapeutics in NSCLC (which include platinum-based drugs, such as cisplatin or carboplatin, often in combination with pemetrexed or other antimetabolites or antimicrotubular agents) as well as with other chemodrugs commonly used for the treatment of carcinomas. Recent preclinical and clinical studies show promising results on combined treatments with ATR inhibitors and cisplatin and/or other drugs in lung cancer or other carcinomas [47–51] in that respect, it will be very interesting to correlate treatment responses with p53 and BOK statuses, respectively. Understanding the broader impact of BOK-mediated uridine metabolism on DNA repair and genomic stability may also open new avenues for targeting metabolic vulnerabilities in cancer therapy. Ultimately, this work sheds light on a novel and actionable pathway in lung cancer, with potential for therapeutic exploration.

## Materials and methods

### Cell culture

Mutant KRAS^G12D^ NSCLC cells modestly but constitutively overexpressing cMyc under the ROSA26 promoter (*lsl-KRas*^*G12D*^;*Rosa26-lsl-MYC*) were derived from primary mouse tumours and kindly provided by Daniel J Murphy (Glasgow, UK) [35]. Cells were cultured in RPMI-1640 StableCell™ medium containing stable glutamine (Merck, Buch, CH) complemented with 10% foetal calf serum (FCS) (SeraLow, PAN-Biotech GmbH, Aidenbach, DE), penicillin (100 U/mL)/streptomycin (100 µg/mL) and 50 µM 2-Mercaptoethanol (all Thermo Fisher Scientific (Schweiz) AG, Reinach, CH). A549 cells (ATCC; authenticated by STR profiling) were cultured in DMEM high glucose GlutaMAX™ medium complemented with 10% FCS, penicillin/streptomycin, and 100 µM sodium pyruvate. Cells were split biweekly by trypsinisation with 0.25% Trypsin-EDTA solution (Merck, Buchs, CH). Cells were cultured in an incubator at 37°C and 5% CO_2_.

### Cell growth quantification with PrestoBlue^TM^-Assay

2×10^3^ cells were seeded in 90 µL of appropriate medium in a 96-well plate. At the respective timepoints, 10 µL of PrestoBlue^TM^ HS cell viability reagent (Thermo Fisher Scientific (Schweiz) AG, Reinach, CH) were added. After 1 h of incubation at 37°C, fluorescence was quantified using a SpectraMax M2 spectrophotometer (Molecular Devices, San José, CA, US). Signal was normalised to the T_0_ signal and plotted as fold increase.

### Comet assay

Comet Assay was performed using the CometAssay Single Cell Gel Electrophoresis Assay (bio-techne, Minneapolis, MN, US) according to the manufacturer instructions. Staining was subsequently performed for 30 min at room temperature, using 100 µL of propidium iodide solution (20 µg/mL). Pictures of 50 randomly chosen nuclei per condition were then taken either by confocal microscopy (Zeiss LSM 800, Zeiss, Jena, DE) or by fluorescent microscopy (Keyence BZ-X810, Keyence Deutschland GmbH, Neu-Isenburg, DE). Signals were then quantified using ImageJ software (V1.53t) developed by Wayne Rasband (National Institutes of Health, USA) and contributors. Experiments were repeated until at least totally 150 nuclei were quantified from at least 3 independent experiments for each condition.

### CRISPR/Cas9 mediated gene editing

The human and mouse *BOK* loci were targeted using CRISPR/Cas9 technology, using lentiCRISPR v2 plasmid (Addgene plasmid #52961), while human *TP53* was targeted using lentiCRISPR v2 (hygro), in which the puromycin resistance cassette was exchanged for a hygromycin resistance cassette. Working guide RNAs to disrupt human BOK have been described previously [27] and the primer sequences for subcloning into LCV2 were as follows:

Fw 5’-**caccg**GTCTGTGGGCGAGCGGTCAA-3’

Rev 5’-**aaac**TTGACCGCTCGCCCACAGAC**c**-3’

To target mouse *Bok* or human *TP53*, we designed and tested three guide RNAs, each, using the publicly available service at http://crispr.mit.edu. The most efficient knockdowns were obtained using the following target sequences:

*TP53:*

Forward: 5’ **caccg**CCCCTTGCCGTCCCAAGCAA-3’

Reverse: 5’ **aaac**TTGCTTGGGACGGCAAGGGG**c**-3’

*Bok*:

Forward: 5’-**caccg**GTCTGTGGGCGAGCGATCAA-3’

Reverse: 5’-**aaac**TTGATCGCTCGCCCACAGAC**c**-3’

HEK293T cells were transfected with the plasmids lentiCRISPR v2, pMD2GVSV-G, psPAX2 using X-tremeGENE HP DNA Transfection Reagent (Roche). Viruses were harvested after 24 h, filter-sterilised and freshly added to semi-confluent cultures of A549 or KRAS-MYC NSCLC cells in the presence of 8 μg/mL polybrene, followed by 3 weeks of selection with 2 µg/mL puromycin or 500 µg/mL of hygromycin.

Successful gene editing was validated by sequencing of the target region, confirming the intended modifications, which resulted in frameshift mutations causing early STOP codons in all cases (see Suppl. Table S2 + Suppl. Fig. S5).

The subcloning of mouse *Bok*^*-/-*^ NSCLC line #1 was performed by limiting dilution, seeding 0.5 cells per well (96-well format, round bottom) on average. After a period of 2 weeks, single colonies of cells were harvested, and BOK-status was verified by western blotting. 3 subclones with complete lack of BOK expression were then pooled to perform the experiments.

### TAT fusion peptides

TAT based fusion peptides spanning the BH3 domain of BOK, or a BH3(AAA) mutant, containing a N-terminal FITC-ahx and C-terminal amide modification, were ordered form ChemPeptide (Shanghai, CN). Amino acid sequences were as follows:

TAT-BH3: GRKKRRQRRRPPQRLAEVCAVLLRLGDELEMIRPSVYR

TAT-BH3(AAA): GRKKRRQRRRPPQRLAEVCAVLAAAGDELEMIRPSVYR

Peptides were diluted down to a 10X concentration in double distilled water and added for 24 h to the cells.

### Colony formation assay & crystal violet staining

1 ×10^4^ A549 cells or 5 ×10^3^ KRAS-MYC NSCLC cells were seeded per well in 6-well plates and let grow for 6 (KRAS-MYC-NSCLC) or 4 days (A549), respectively. Cells were fixed for 10 min at 4°C using a 4% (v/v) paraformaldehyde solution in double distilled water. Then the paraformaldehyde was removed, and the cells were stained with 0.5% crystal violet solution dissolved in 20% methanol for 1 min at RT and subsequently washed four times with double distilled water. The wells were then scanned using an Epson perfection 4490 scanner and signals quantified using ImageJ software (V1.53t)

### Quantification of cell death by flow cytometry

Cells (adherent and floating) were harvested and pooled into 5 mL FACS tubes. Cells were pelleted at 490 x g for 5 min at room temperature. Samples were then stained with Atto633-AnnexinV diluted in FACS Buffer (150 mM NaCl, 4 mM KCl, 2.5 mM CaCl_2_, 1 mM MgSO_4_, 15 mM HEPES pH 7.2, 2% FCS and 10 mM NaN_3_) for 20 min on ice in the dark. Cells were washed once and resuspended in FACS buffer containing 2 µg/mL propidium iodide. Viability of cells was quantified by flow cytometry using BD FACS Lyric or Verse (Becton, Dickinson and Company, Franklin Lake, NJ, US) and results were analysed using FlowJo V10.5.3 (FloJo, Ashland, OR, US).

### Quantification of DNA damage by pH2A.X

Cells were seeded on glass bottom CellView cell culture slides (Greiner Bio-One VACUETTE Schweiz GmbH, CH). Cells were fixed in 4% (v/v) paraformaldehyde for 10 min at 4°, washed in PBS and permeabilized in 0.05% saponin solution for 5 min at room temperature and subsequently incubated in -20°C cold acetone for 10 min. After a blocking step for 15 min at room temperature in blocking buffer (1% normal goat serum, 1% BSA in PBS), cells were incubated with primary rabbit monoclonal anti-p(Ser139)-H2A.X antibody (clone 20E3, Cell Signaling Technology, Danvers, MA, US) 1:400 in blocking buffer for 1 h at room temperature, followed by incubation (90 min, RT, 1:400 in blocking buffer) using Alexa647-conjugated goat anti-rabbit F(ab')2 IgG(H + L) secondary antibody (Jackson ImmunoResearch, West Grove, PA, US). Postfixation in 4% (v/v) paraformaldehyde containing DAPI (1:5’000) was then performed for 10 min at room temperature.

Cells were analysed using a Keyence fluorescence microscope (Keyence BZ-X810, Keyence Deutschland GmbH, Neu-Isenburg, DE). Images were acquired at 600x magnification and consistent exposure times for every experiment. pH2A.X signals of 50 nuclei per condition were quantified using ImageJ software (V1.53t). Nuclei were marked for analysis using the DAPI staining, then total pH2A.X fluorescence was measured and background fluorescence was subtracted. The following formula was used for the calculation:

Signal (pH2A.X) = total fluorescence(nucleus)-area(nucleus)*background signal

Results were normalised to the isotype control condition. The experiment was repeated until a minimum of totally 150 nuclei from at least 3 independent experiments were quantified.

### Western blotting

Total protein lysates were prepared in hot H8 lysis buffer (20 mM Tris-HCl pH 7.5, 2 mM EGTA,2 mM EDTA, 1% SDS and 50 mM DTT) and homogenisation using 30 G syringes. Protein concentrations were determined using NanoDrop prior to boiling in Laemmli buffer. 12.5% SDS-PAGE electrophoresis gels were cast, and equal amounts of sample were loaded per well. PageRuler protein ladder (Thermo Fisher Scientific (Schweiz) AG, Reinach, CH) was included as molecular weight marker. After electrophoresis was performed the gels were then transferred onto a PVDF membrane (Immobilon-F, Merck, Buchs, CH). Blocking was performed for 1 h in blocking buffer appropriate for the antibody used. Incubation with primary antibody was performed overnight. Primary antibodies used were rabbit monoclonal anti-BOK (Rab 1-5) [9], rabbit monoclonal anti-pH2A.X (Ser139) (clone 20E3, Cell Signaling Technology 9718), rabbit monoclonal anti-PUMA (clone E2P7G; Cell Signaling Technology 98672), rabbit polyclonal anti-p21 (Santa Cruz sc-397), rabbit polyclonal anti-p53 (Santa Cruz sc-6243), mouse monoclonal anti-CHK1 (clone 2G1D5; Cell Signaling Technology 2360), rabbit monoclonal anti-pCHK1 (Ser317) (clone D12H3; Cell Signalling 12302), mouse monoclonal anti-actin (Becton Dickinson Cat. No. 612656). Membranes were washed three times in PBS + 0.1% Tween-20 and incubated for 90 min at RT with species-specific HRPO-conjugated secondary antibodies (Jackson ImmunoResearch, West Grove, PA, US) diluted 1:20’000 in PBS/Tween and subsequently washed three times. Immobilon Forte Western HRPO substrate (Merck, Buchs, CH) was added and chemiluminescent signals acquired on a LI-COR Odyssey XF or Fc System and analysed using Image Studio Lite V5.2.5 Software (all LI-COR Biosciences GmbH, Bad Homburg, DE).

Uncropped images of the western blot membranes, which show all the data including control lanes and replicates, are available in the Supplementary Online Materials for the article.

### Statistical analyses

Two-way ANOVA and T-Tests were performed using GraphPad Prism 8 V8.0.1 (244). Kruskal-Wallis analysis and Mann-Whitney tests were performed using R Version 4.2.1. Bonferroni correction was either performed as part of the R script (for the Mann-Whitney tests) or manually by Microsoft Excel Version 2506. All error bars represent mean +/- standard deviation. *: *p* ≤ 0.05; **: *p* ≤ 0.01; ***: *p* ≤ 0.001; ****: *p* ≤ 0.0001

## Supplementary information

Online Supplementary Material

Raw western blot data

## Data Availability

All data generated or analysed during this study are included in this published article and its supplementary information files.
