# Supplementary material for: Loss of BOK increases vulnerability of p53 deficient non-small cell lung cancer cells to ATR inhibition through its role in uridine metabolism
Source: Cell Death Differ. 2026 Jan 29;33(9):1878–90. doi: 10.1038/s41418-026-01666-0 (PMC13545241; doi:10.1038/s41418-026-01666-0)
Supplement: Supplementary file 1 [file 41418_2026_1666_MOESM1_ESM.pdf]

ONLINE SUPPLEMENTARY MATERIAL

JeanRichard P *et al,* Loss of BOK increases vulnerability of p53 deficient non-small cell lung cancer cells to ATR inhibition through its role in uridine metabolism

Supplementary Figures

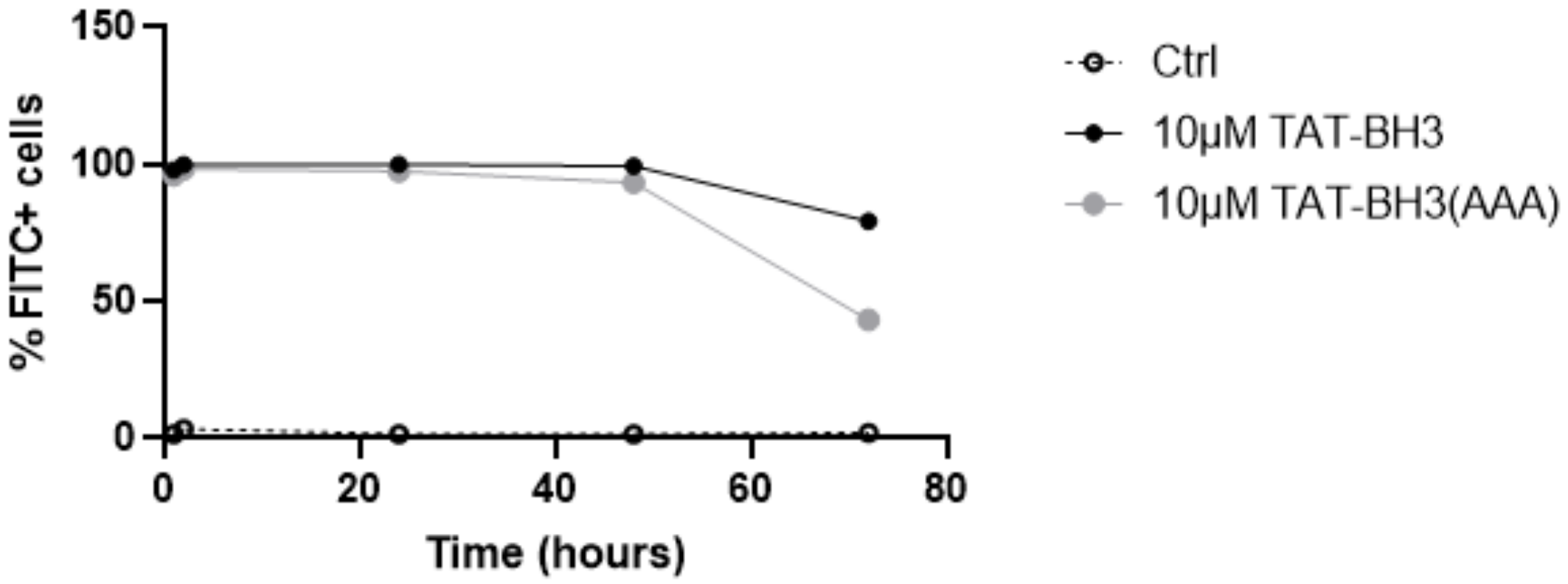

Supplementary Figure S1: Percent of FITC positive cells over time post treatment with 10 µM FITC-TAT-BH3 or 10 µM FITC-TAT-BH3(AAA).

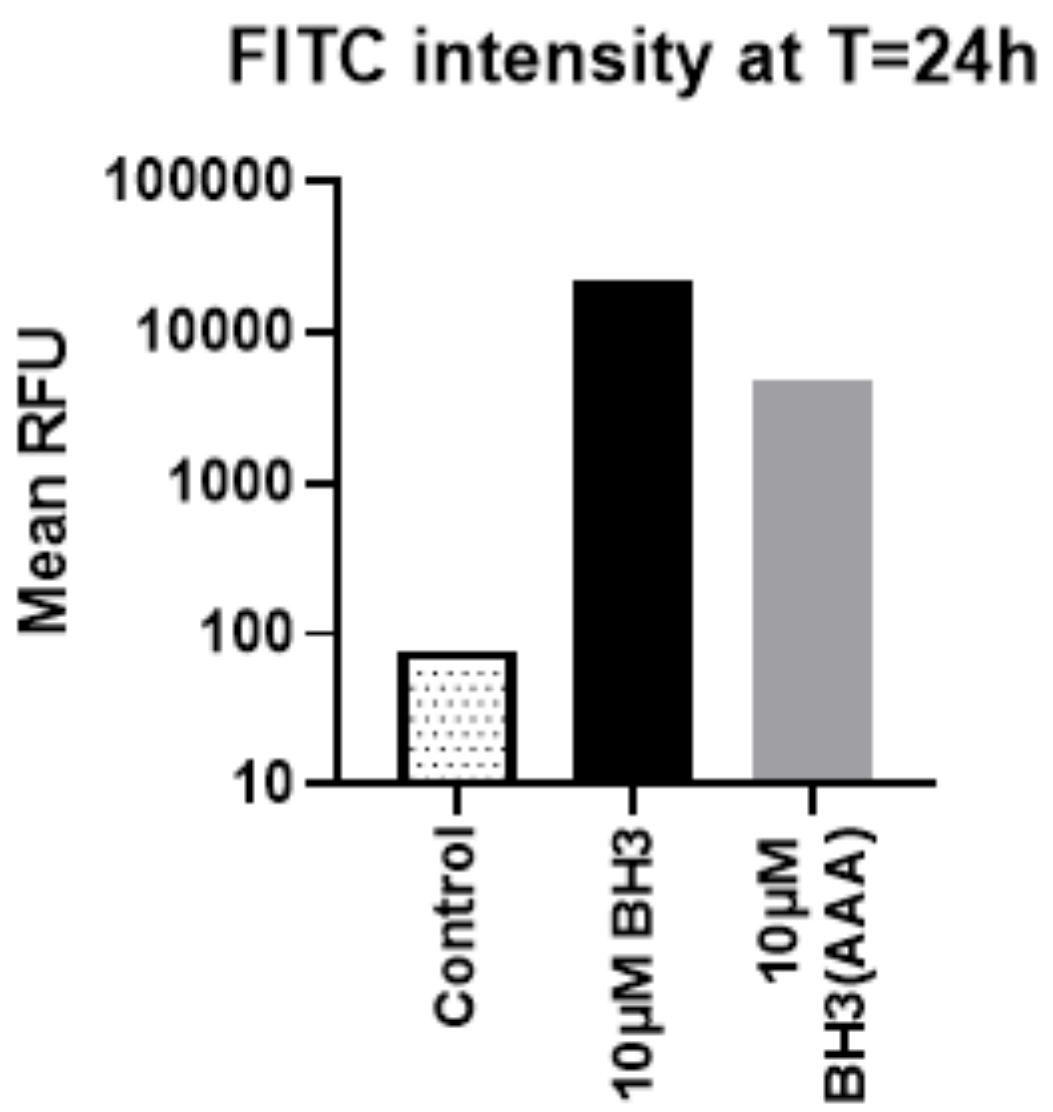

Supplementary Figure S2: Mean RFU values 24h post treatment with 10 µM FITC-TAT-BH3 or 10 µM FITC-TAT-BH3(AAA).

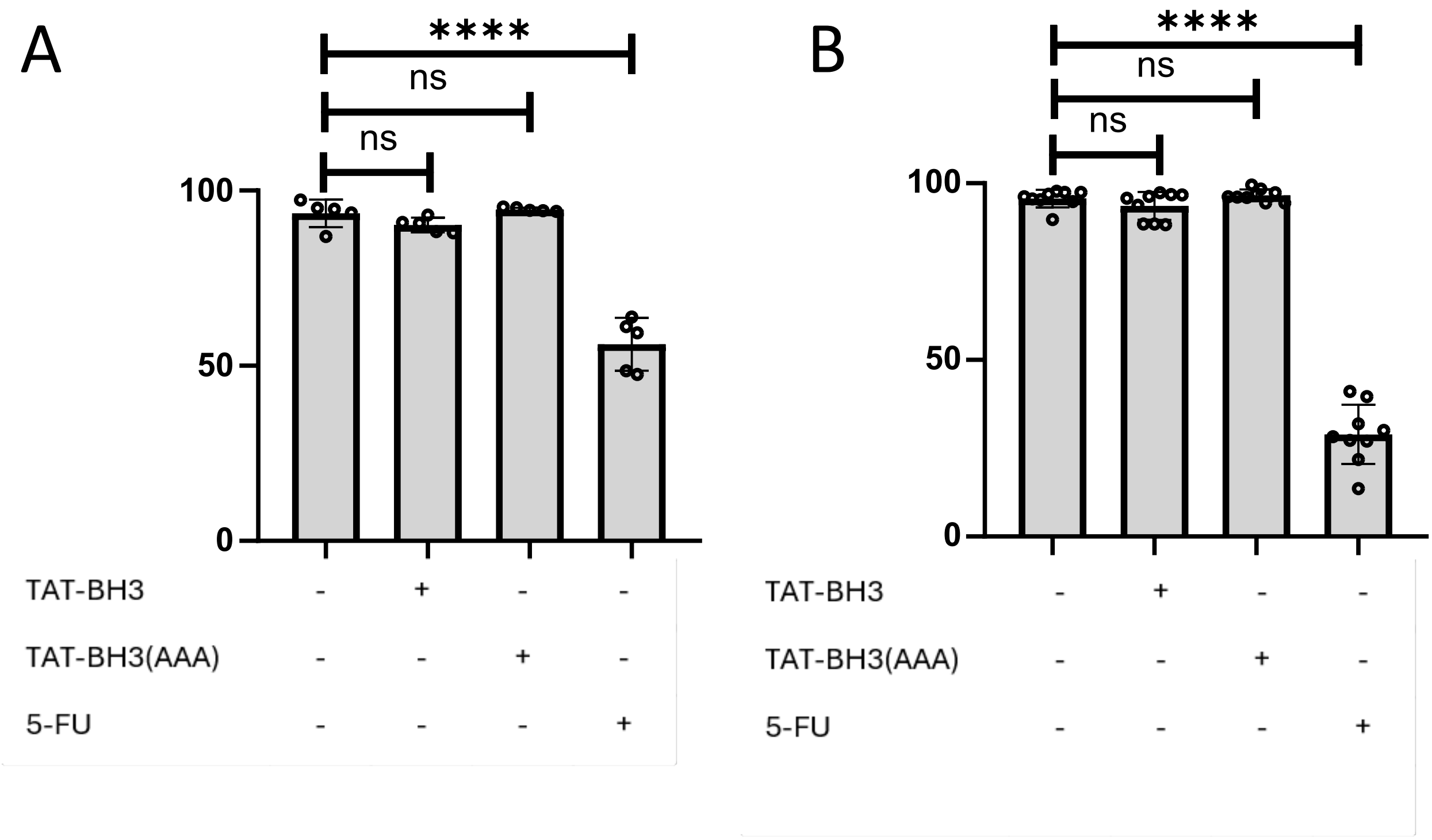

Supplementary Figure S3: Cell survival was quantified by flow cytometry using AnnexinV/PI exclusion. Survival was defined as the percentage of AnnexinV/PI double negative cells. Cells were treated for 24 hours with 10 μM FITC-TAT-BH3, 10 μM FITC-TAT-BH3(AAA) or 72 hours with 100 μg/mL 5-FU (N ≥ 5). **A:** A549 cells **B:** KRAS-MYC NSCLC cells. Statistical analysis was done by TWO-way ANOVA, followed by pairwise Bonferroni-adjusted T-tests. *: p ≤ 0.05; **: p ≤ 0.01; ***: p ≤ 0.001; ****: p ≤ 0.0001. Error bars represent mean +/- standard deviation.

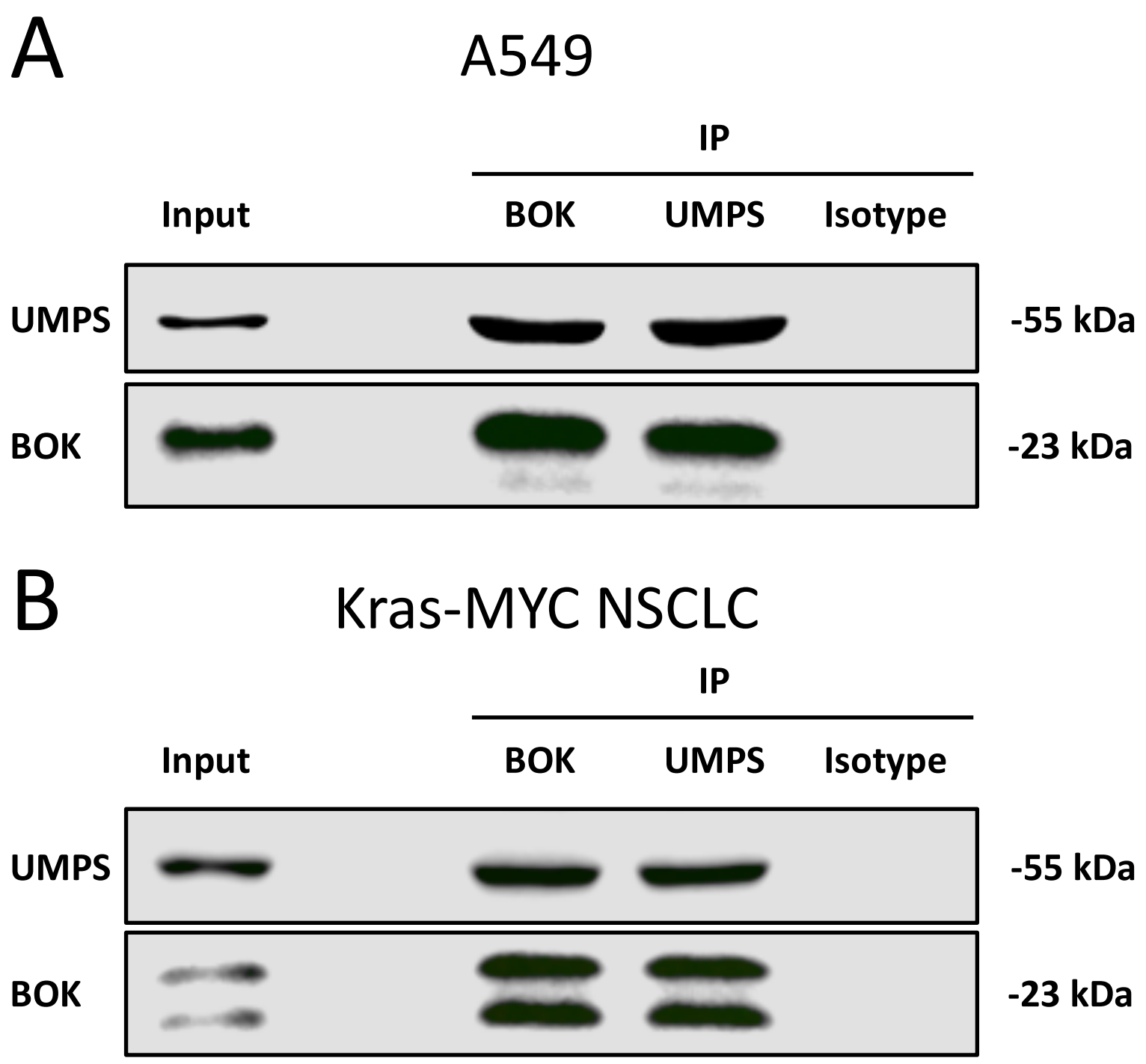

Supplementary Figure S4: Co-immunoprecipitation of endogenous BOK and UMPS. **A:** A549 cells **B:** KRAS-MYC NSCLC cells.

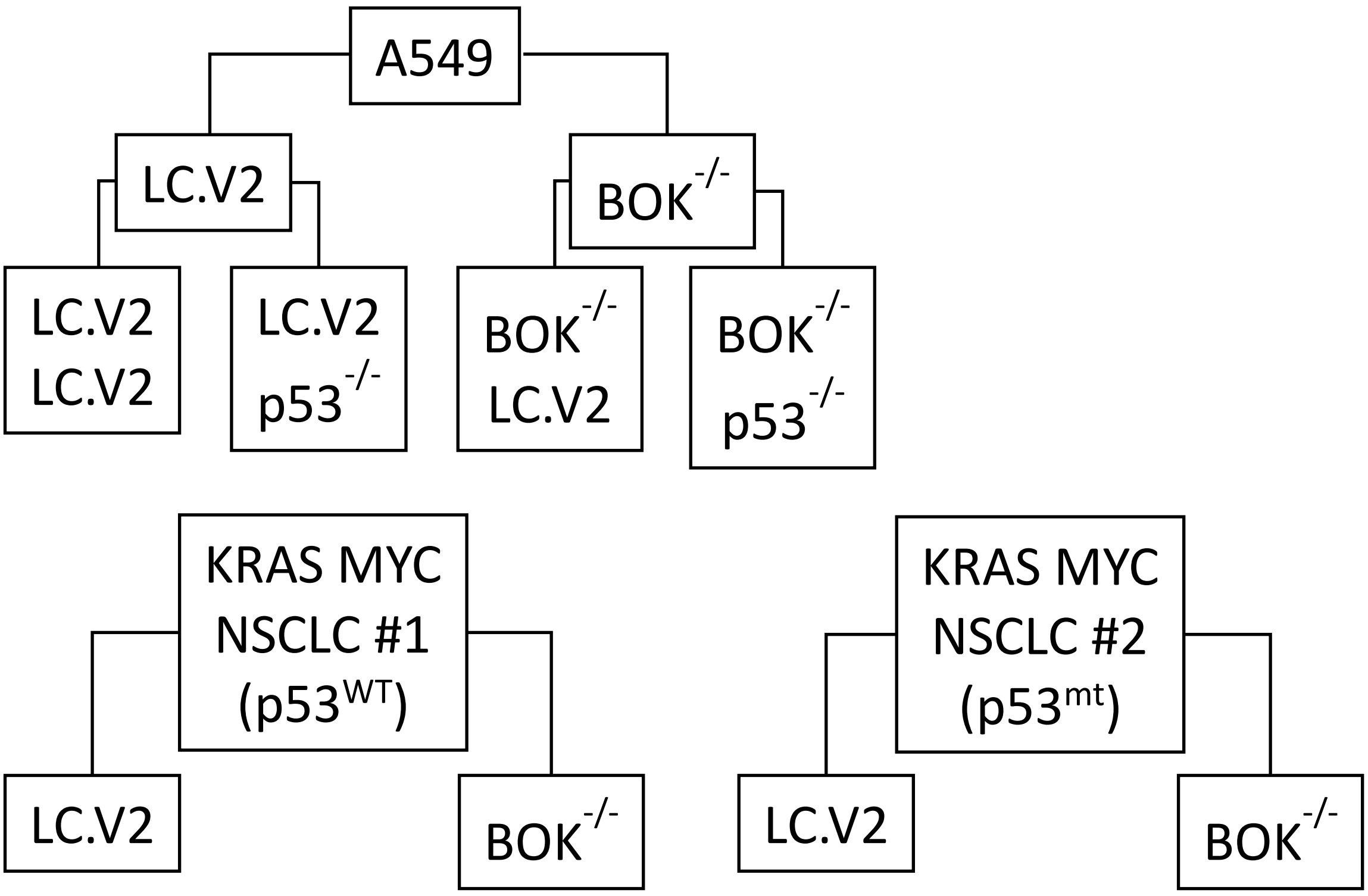

Supplementary Figure S5: Cell line relations. Scheme depicting the generation of A549 and KRAS MYC cell lines lacking BOK and/or p53.

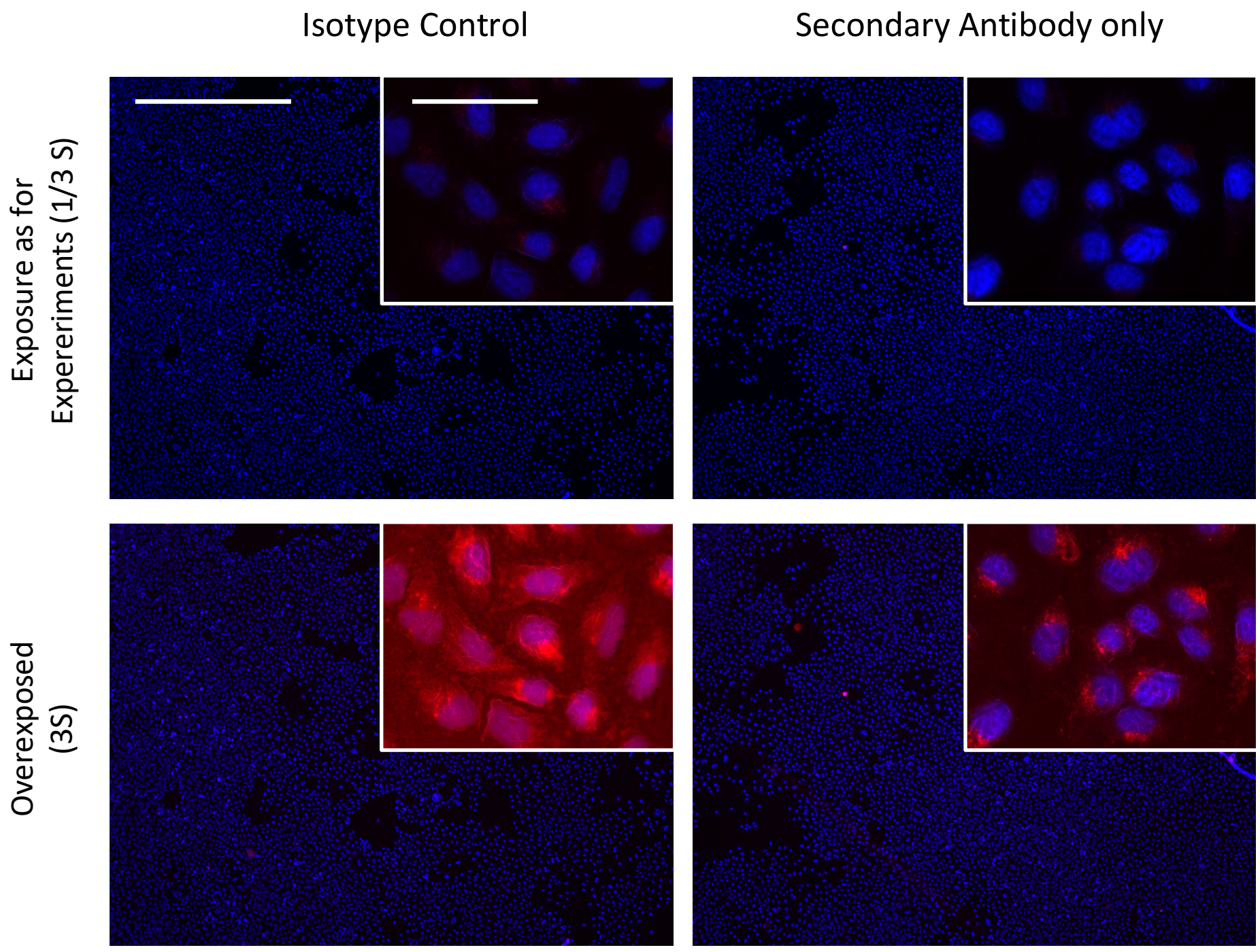

Supplementary Figure S6: Isotype (top left) and secondary antibody only (top right) control of pH2A.X quantification. Both conditions were excited with the same fluorescence intensity that was used in the pH2A.X quantification experiments (1/3 sec). In both cases, the presence of secondary antibody was verified by overexposure (3 sec) (bottom row).

Supplementary Table S1. List of mouse TP53 mutations found by sequencing in KRAS-MYC NSCLC cell line #2

Mutation	Domain
C40S	Transactivation domain
L45V	Transactivation domain
V50G	Transactivation domain
R62Q	Transactivation domain
S64V	proline rich domain
V73G	proline rich domain
L90P	proline rich domain
S91A	proline rich domain
117–122 (KSVMCT→NLLL)	DNA-binding domain
N128H	DNA-binding domain
V140G	DNA-binding domain
A150P	DNA-binding domain
Y160F	DNA-binding domain
M166I	DNA-binding domain
T167L	DNA-binding domain
R178C	DNA-binding domain
P200A	DNA-binding domain
R206K	DNA-binding domain
S212R	DNA-binding domain
N236S	DNA-binding domain
M243I	DNA-binding domain
R279W	DNA-binding domain
R280S	DNA-binding domain

R287P	DNA-binding domain
L292P	DNA-binding domain
E295D	Hinge Domain
P297T	Hinge Domain
303–315 (RALPTCTSASPPQ→ ASAAHLRSASPPPKK)	Oligomerisation domain
P319T	Oligomerisation domain
E323N	Oligomerisation domain
T326S	Oligomerisation domain
K328N	Oligomerisation domain
K333N	Oligomerisation domain
E336Y	Oligomerisation domain
L341V	Oligomerisation domain
E346K	Oligomerisation domain
A350V	Oligomerisation domain
E354K	Oligomerisation domain
S356C	C-terminal domain
R360M	C-terminal domain
H362Q	C-terminal domain
S364T	C-terminal domain
G371D	C-terminal domain
Q372L	C-terminal domain
S373L	C-terminal domain
R376P	C-terminal domain

Supplementary Table S2. CRISPR/Cas9 mediated gene editing:

A549 BOK$^{-/-}$

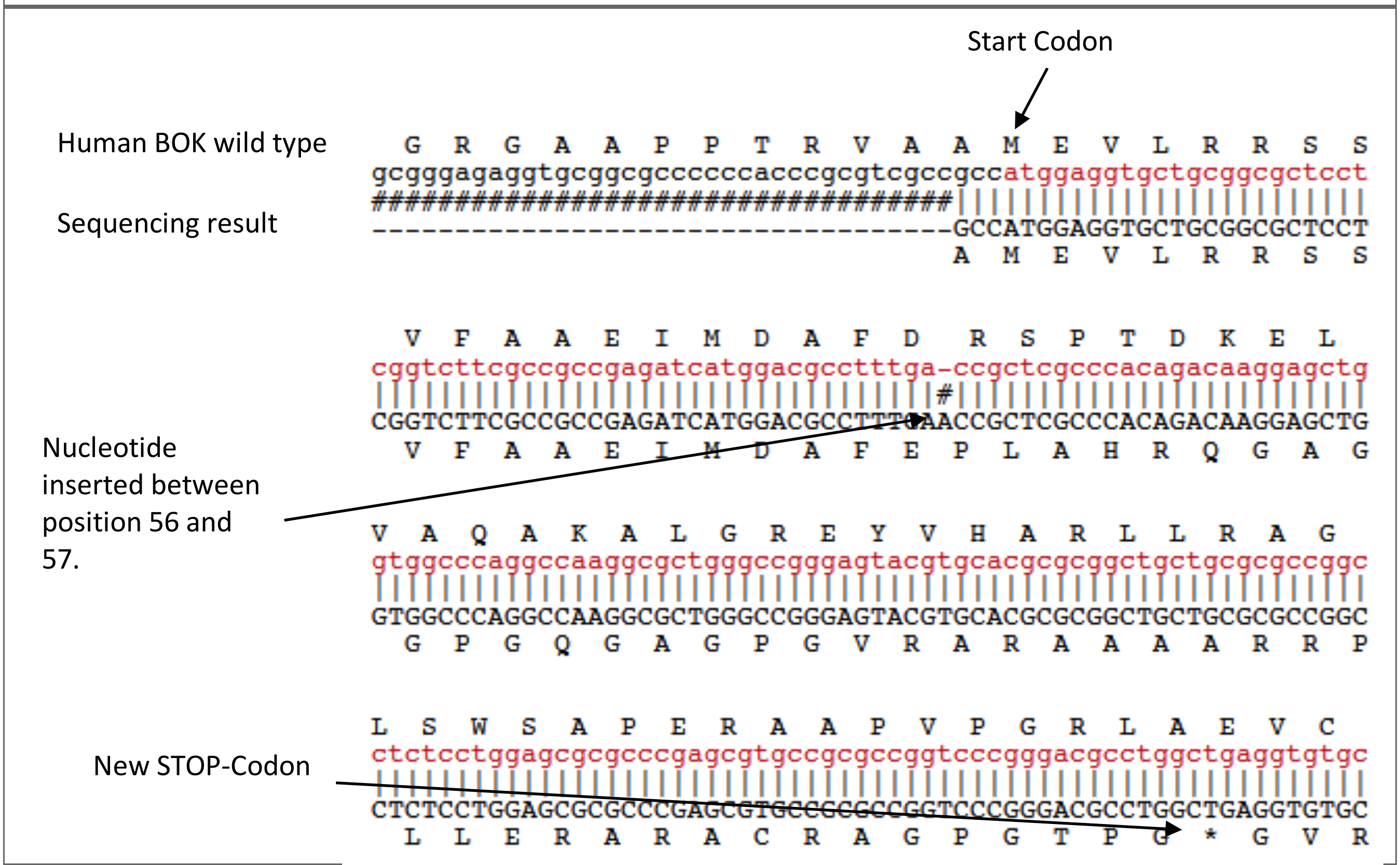

A549 LC.V2 p53$^{-/-}$

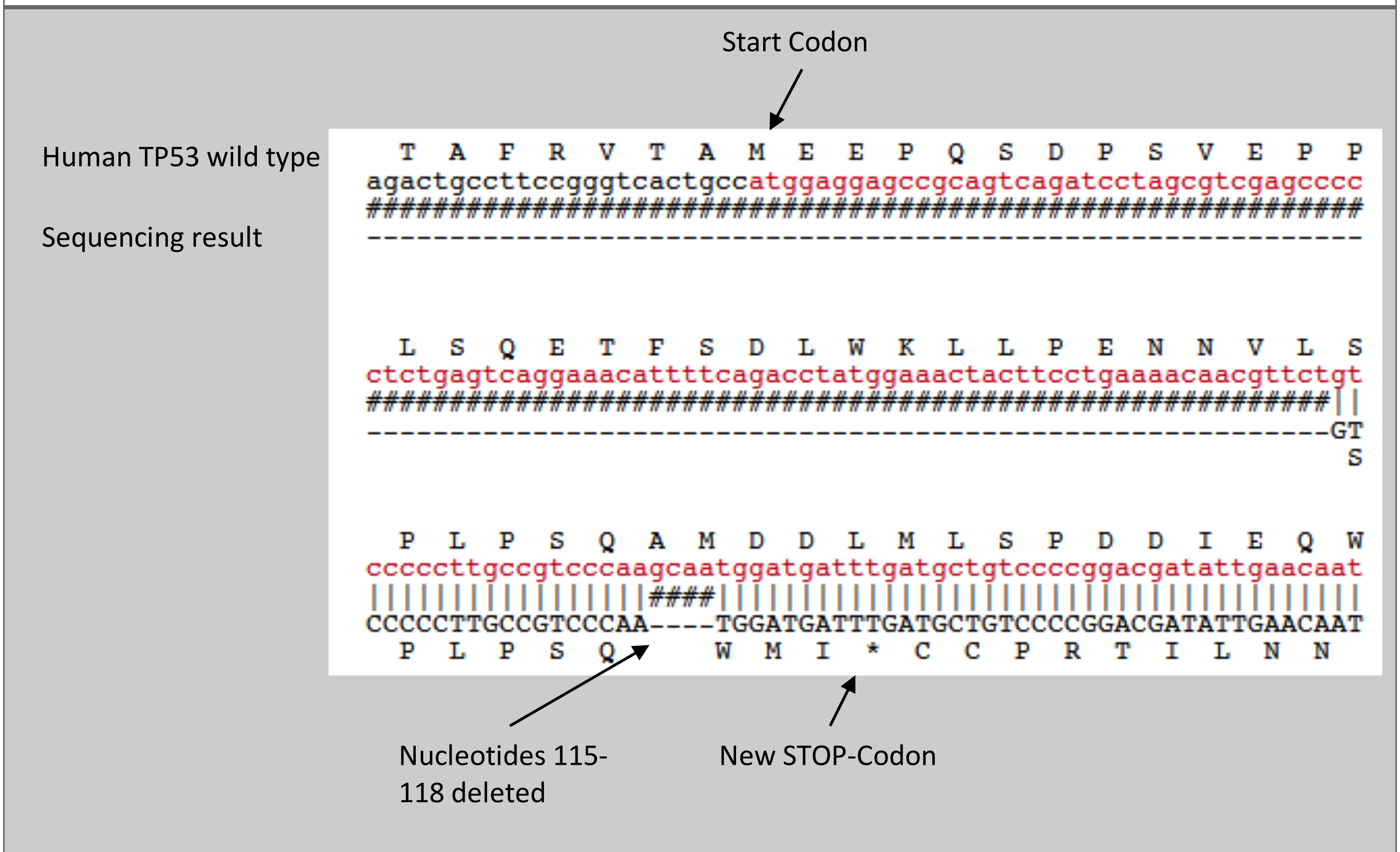

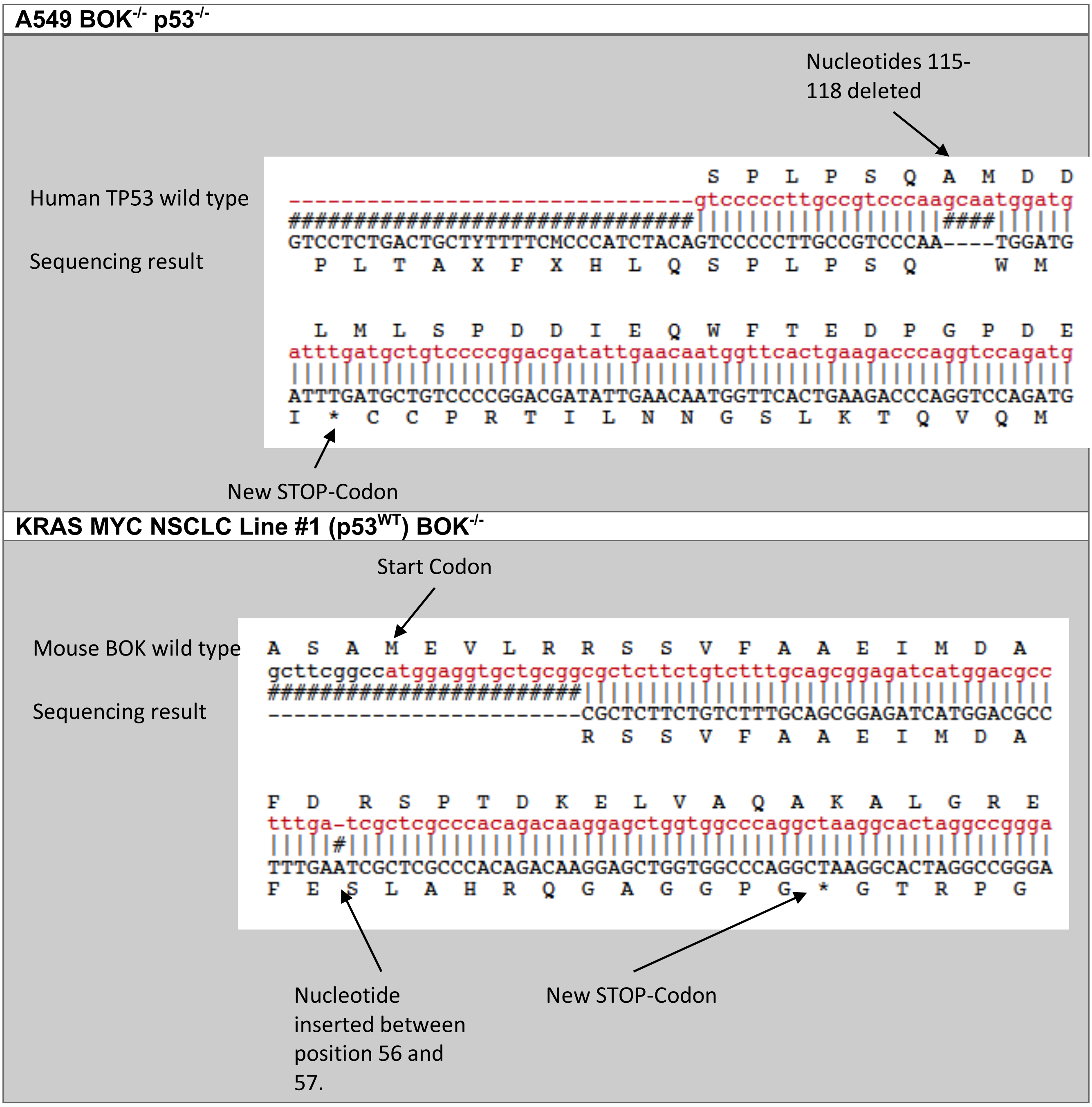
A549 BOK-/- p53-/-
Nucleotides 115-118 deleted
Human TP53 wild type
Sequencing result
S P L P S Q A M D D
--------------------------------gtccccttgccgtcccaagcaatggatg
GTCCTCTGACTGCTYTTTTCMCCCATCTACAGTCCCCCTTGCCGTCCCAA----TGGATG
P L T A X F X H L Q S P L P S Q W M
L M L S P D D I E Q W F T E D P G P D E
atttgatgctgtccccggacgatattgaacaatggttcactgaagacccaggtccagatg
ATTTGATGCTGTCCCCGGACGATATTGAACAATGGTTCACTGAAGACCCAGGTCCAGATG
I * C C P R T I L N N G S L K T Q V Q M
New STOP-Codon
KRAS MYC NSCLC Line #1 (p53WT) BOK-/-
Start Codon
Mouse BOK wild type
Sequencing result
A S A M E V L R R S S V F A A E I M D A
gcttcggccatggaggtgctgcggcgctcttctgtctttgcagcggagatcatggacgcc
------------------------CGCTCTTCTGTCTTTGCAGCGGAGATCATGGACGCC
R S S V F A A E I M D A
F D R S P T D K E L V A Q A K A L G R E
tttga-tcgctcgcccacagacaaggagctggtggcccaggctaaggcactaggccggga
TTTGAATCGCTCGCCCACAGACAAGGAGCTGGTGGCCCAGGCTAAGGCACTAGGCCGGGA
F E S L A H R Q G A G G P G * G T R P G
Nucleotide inserted between position 56 and 57.
New STOP-Codon

KRAS MYC NSCLC Line #2 (p53^mt^) BOK^-/-^

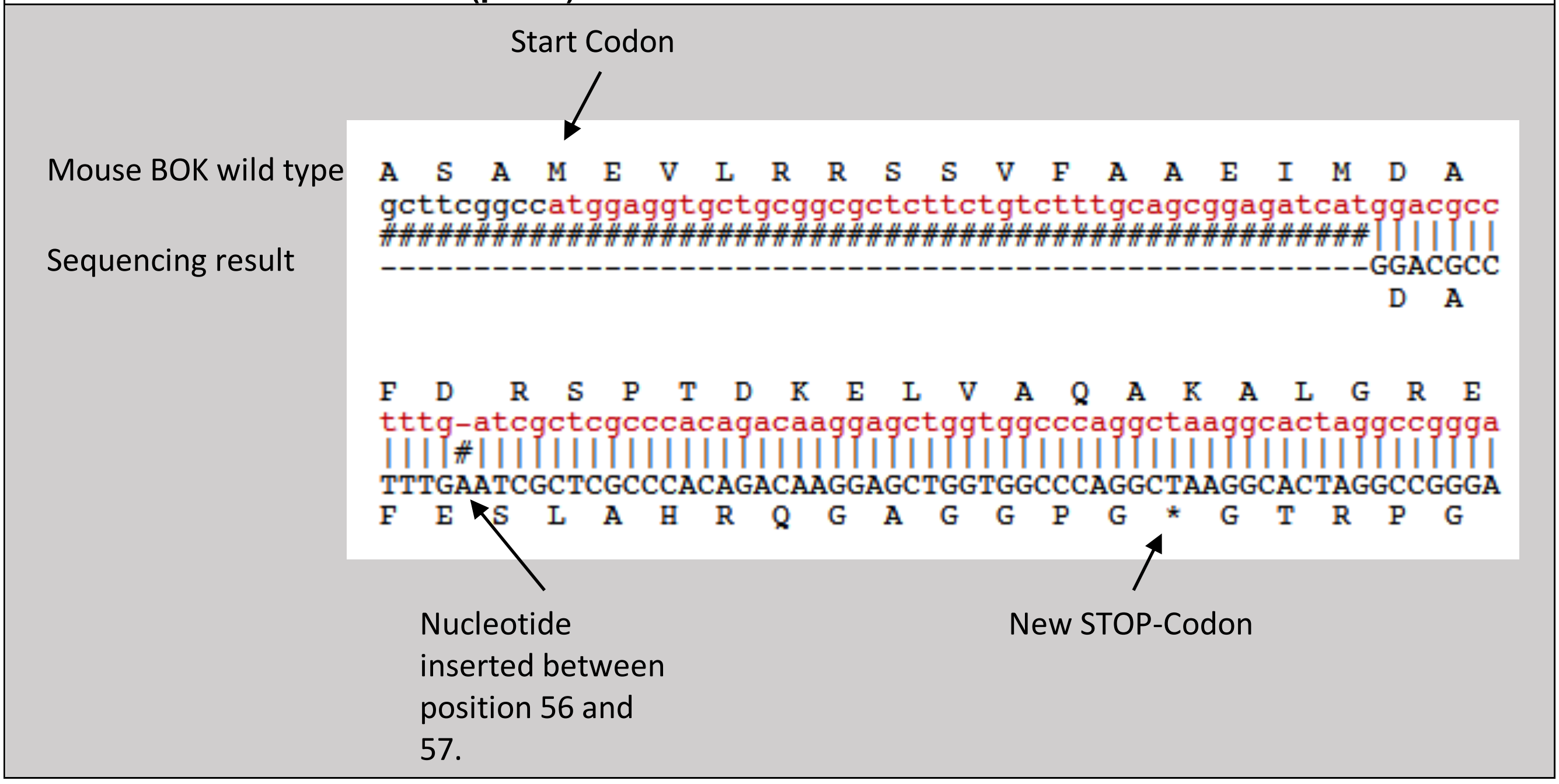

Supplemental Materials

Immunoprecipitation

To immunoprecipitate endogenous UMPS or BOK, 1 x 10^7 A549 or 1 x 10^7 KRAS-MYC NSCLC cells were lysed in 500 µL IP lysis buffer (50 mM Tris/HCl pH 7.4, 150 mM NaCl, 1mM EDTA, 1% Triton X-100, complete protease inhibitor cocktail (Roche Diagnostics) and 1 µg/mL pepstatin) for 30 min on ice and lysates cleared by centrifugation. Lysates were precleared using 25 µl of a 50% slurry of Protein G Sepharose™ 4 Fast Flow beads (Merck, Buch, CH) for 1 h at 4°C on a rotating wheel and a fraction removed as INPUT. Lysates were then incubated with 3 µg of rabbit anti-UMPS (GTX114872, GeneTex, Irvine, CA, US), 6 µg of rabbit monoclonal anti-BOK (RabMab-1-5, in-house (1)), or 6 µg of rabbit IgG isotype control, for 2 h on ice. 50 µl of protein G sepharose beads (50% slurry) were added and the mixture incubated at 4 °C o/n on a rotating wheel. Beads were washed three times in IP lysis buffer and boiled for 5 min in 50 µl of 2x Lämmli buffer containing 100 mM DTT.

References

1. Ke, F, Voss, A, Kerr, JB, O'Reilly, LA, Tai, L, Echeverry, N, Bouillet, P, Strasser, A, and Kaufmann, T. BCL-2 family member BOK is widely expressed but its loss has only minimal impact in mice. Cell Death Differ. 2012;19:915-925.
