# Supplementary material for: Loss of BOK increases vulnerability of p53 deficient non-small cell lung cancer cells to ATR inhibition through its role in uridine metabolism
Source: Cell Death Differ. 2026 Jan 29;33(9):1878–90. doi: 10.1038/s41418-026-01666-0 (PMC13545241; doi:10.1038/s41418-026-01666-0)
Supplement: Supplementary file 2 [file 41418_2026_1666_MOESM2_ESM.pdf]

Uncropped western blots

JeanRichard P *et al*, Loss of BOK increases vulnerability of p53 deficient non-small-cell-lung carcinoma cells to ATR inhibition through its role in uridine metabolism

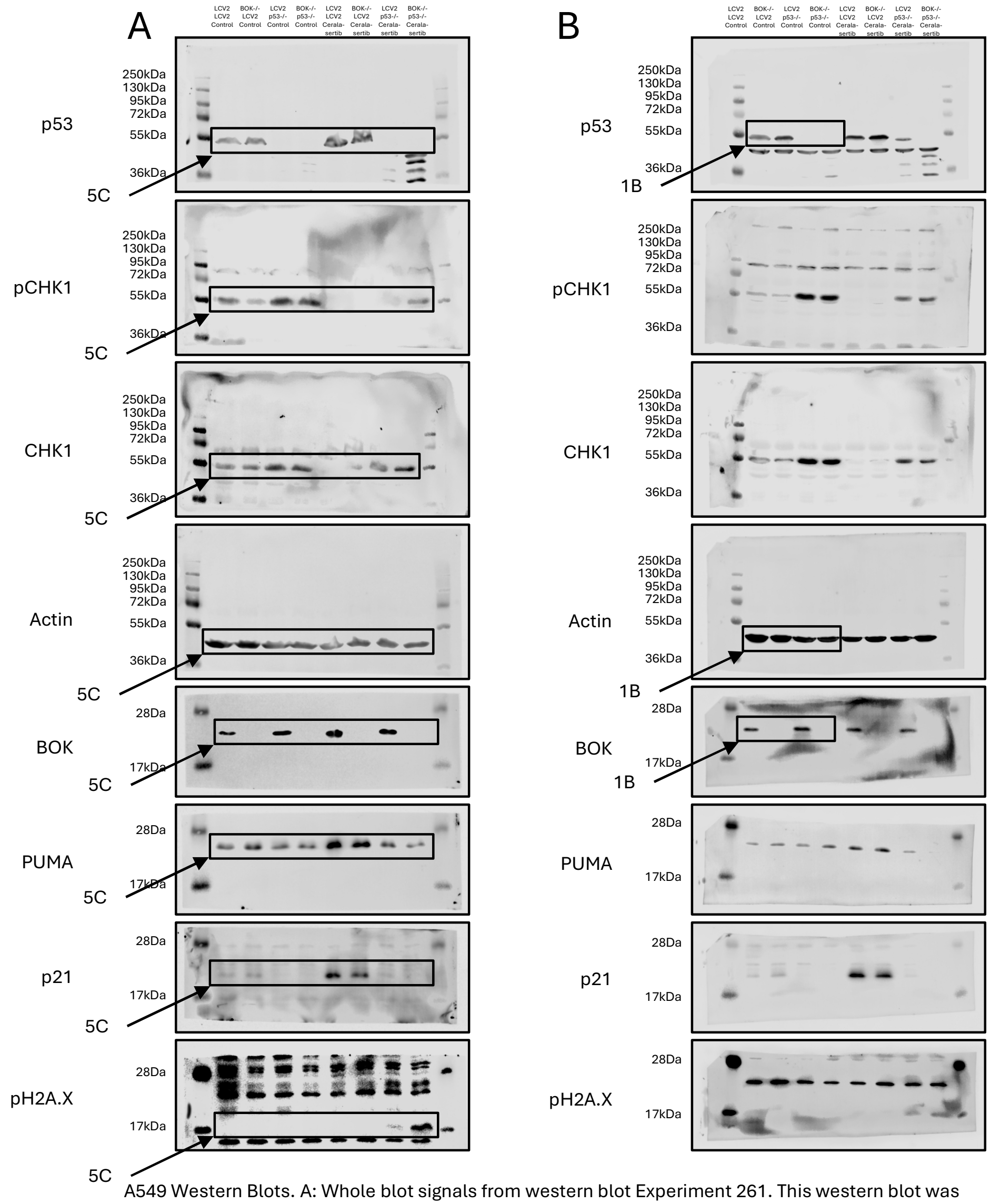

A549 Western Blots. A: Whole blot signals from western blot Experiment 261. This western blot was used as a representative blot in Figure 5C. B: Whole blot signals from western blot Experiment 265. This western blot was used as a representative blot in Figure 1B. All conditions are A549 cells. The PVDF membrane was cut at approximately 30 kDa.

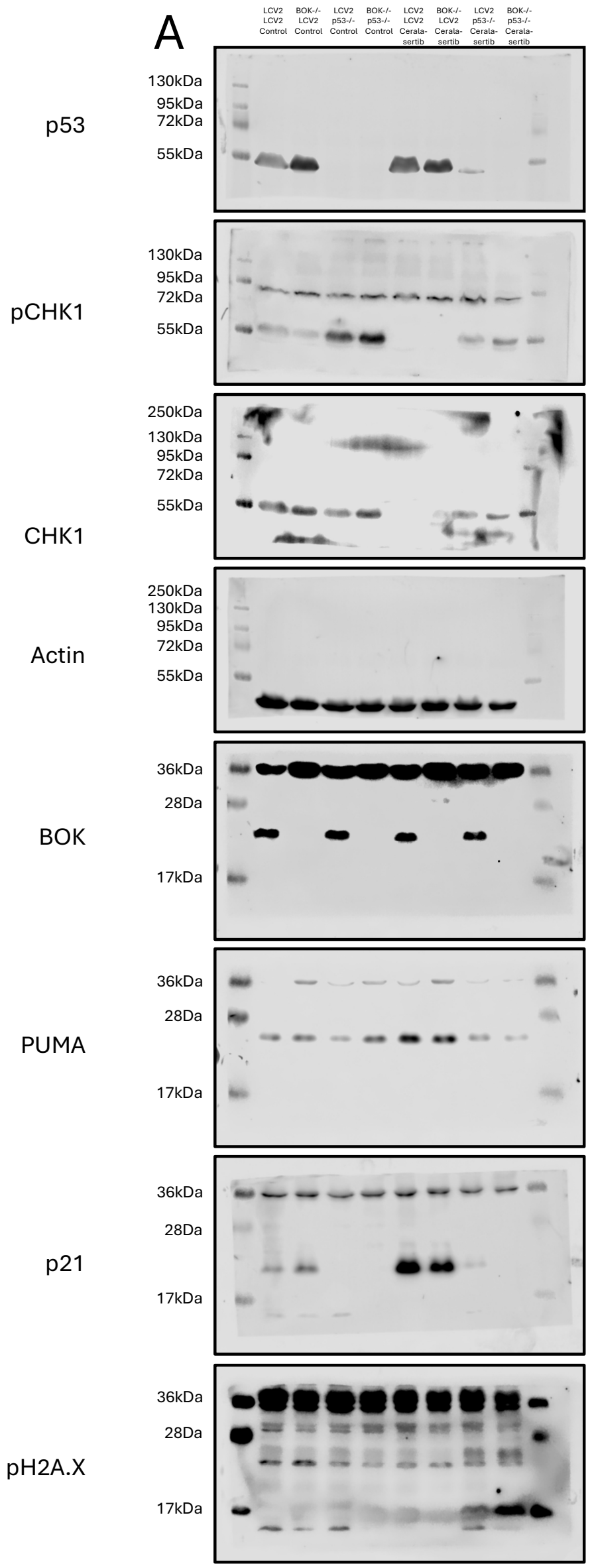

A549 Western Blot. A: Whole blot signals from western blot Experiment 270. All conditions are A549 cells. The PVDF membrane was cut at approximately 40 kDa.

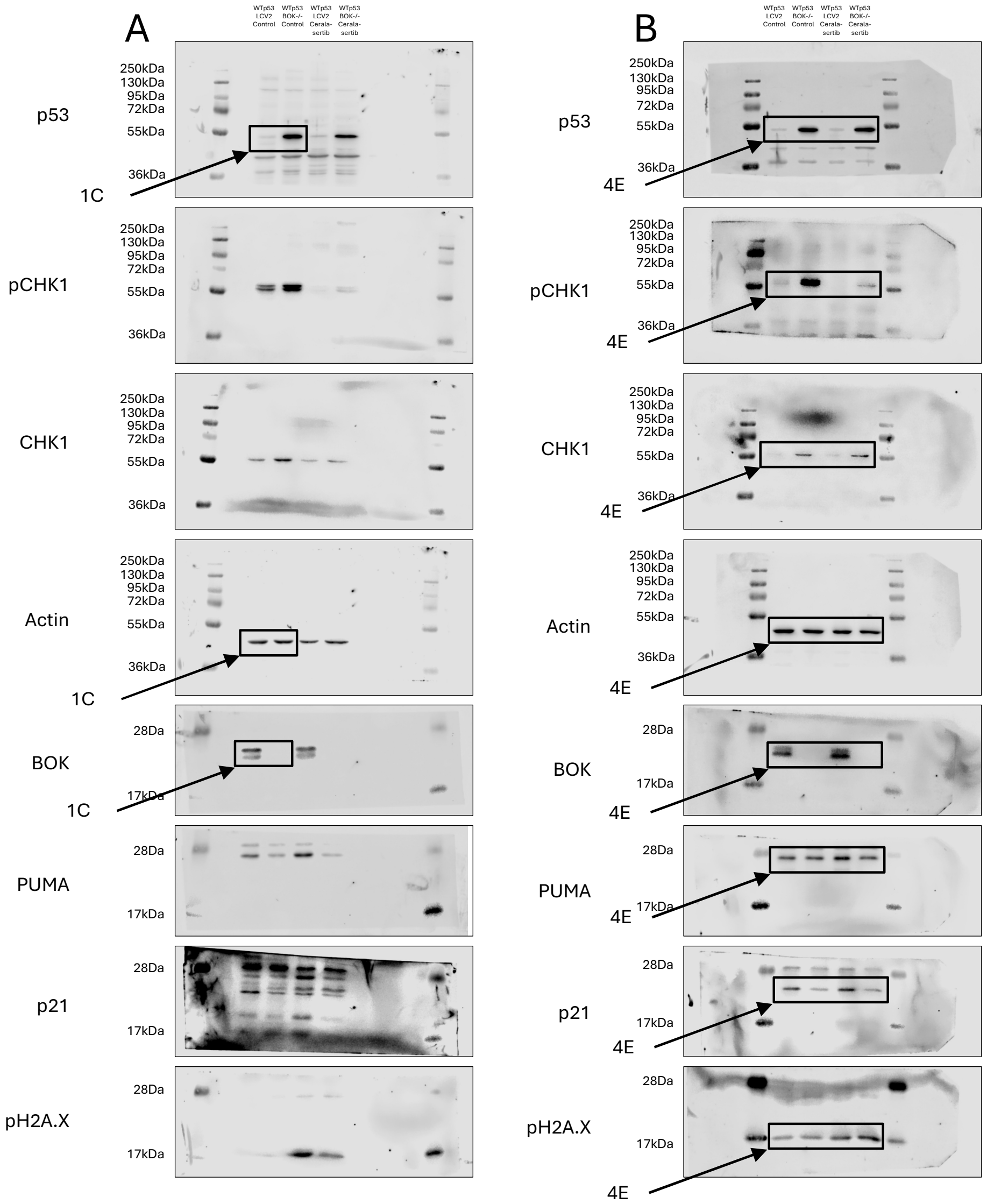

p53[WT] NSCLC Western Blots. A: Whole blot signals from western blot Experiment 286. This western blot was used as a representative blot in Figure 1C. B Whole blot signals from western blot Experiment 291. This western blot was used as a representative blot in Figure 4E. All conditions are NSCLC cells. The PVDF membrane was cut at approximately 30 kDa.

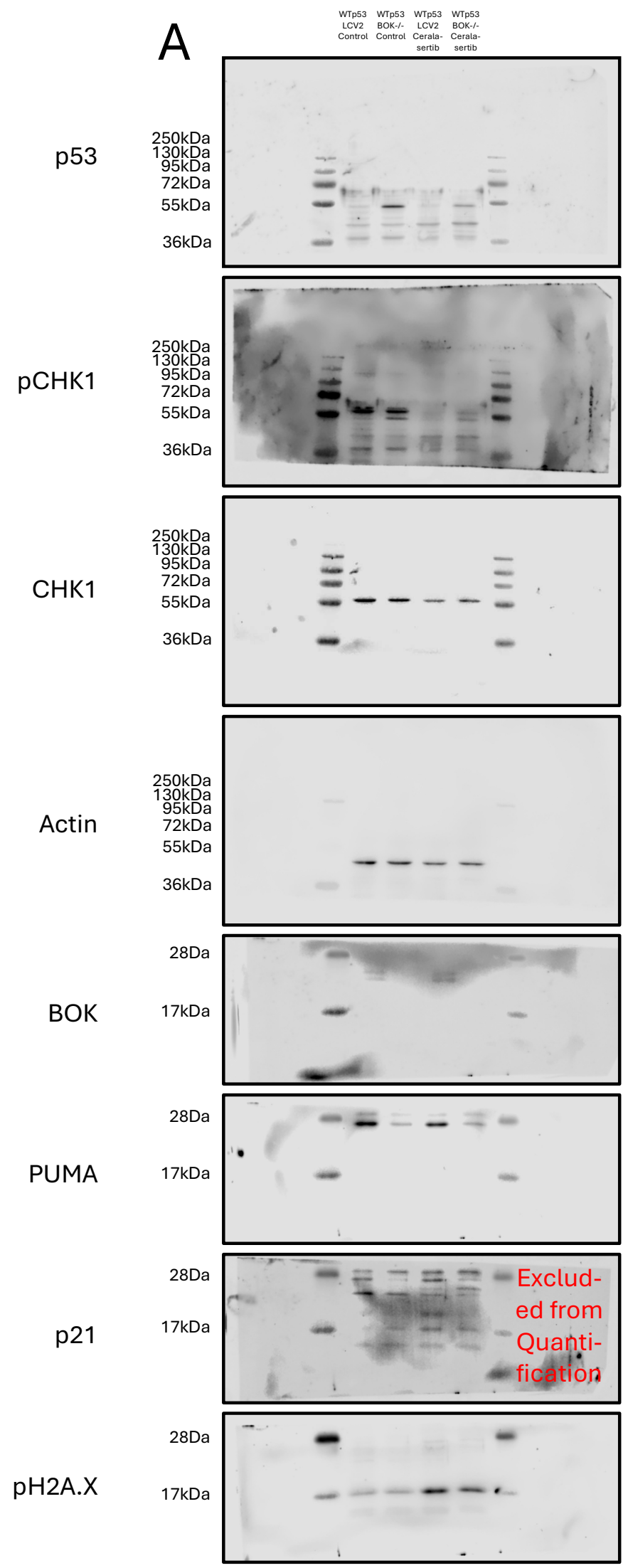

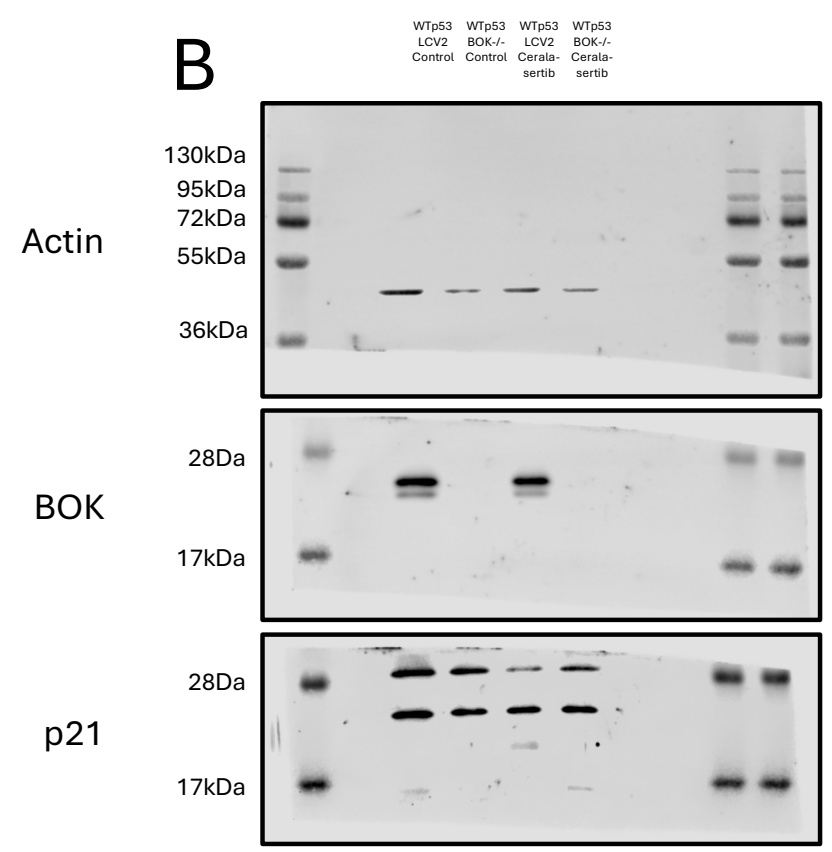

p53[WT] NSCLC Western Blots. A: Whole blot signals from western blot Experiment 309. B: Whole blot signals from western blot Experiment 403. All conditions are NSCLC cells. The PVDF membrane was cut at approximately 30 kDa.

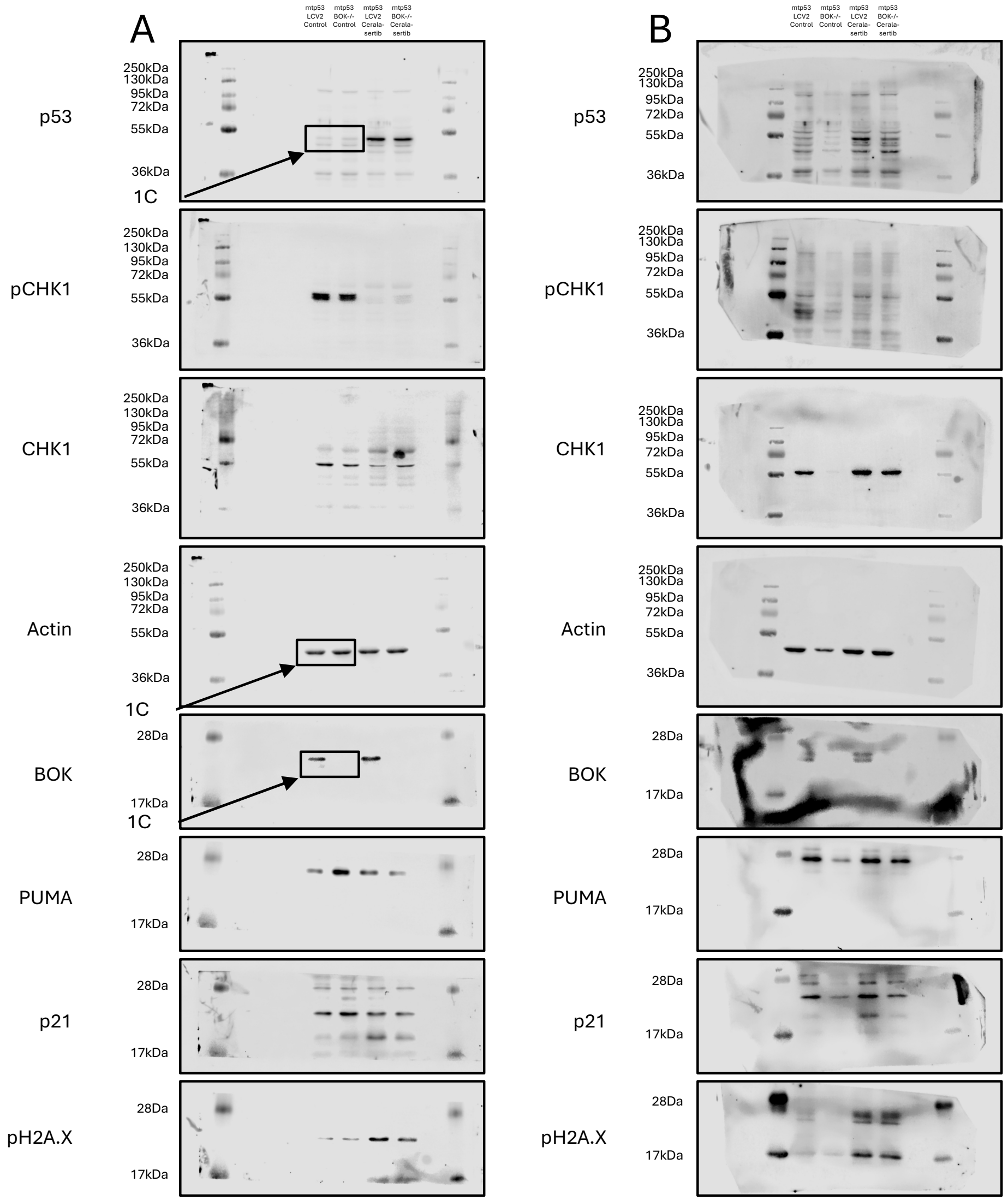

p53[mt] NSCLC Western Blots. A: Whole blot signals from western blot Experiment 287. This western blot was used as a representative blot in Figure 1C. B: Whole blot signals from western blot Experiment 292. All conditions are NSCLC cells. The PVDF membrane was cut at approximately 30 kDa.

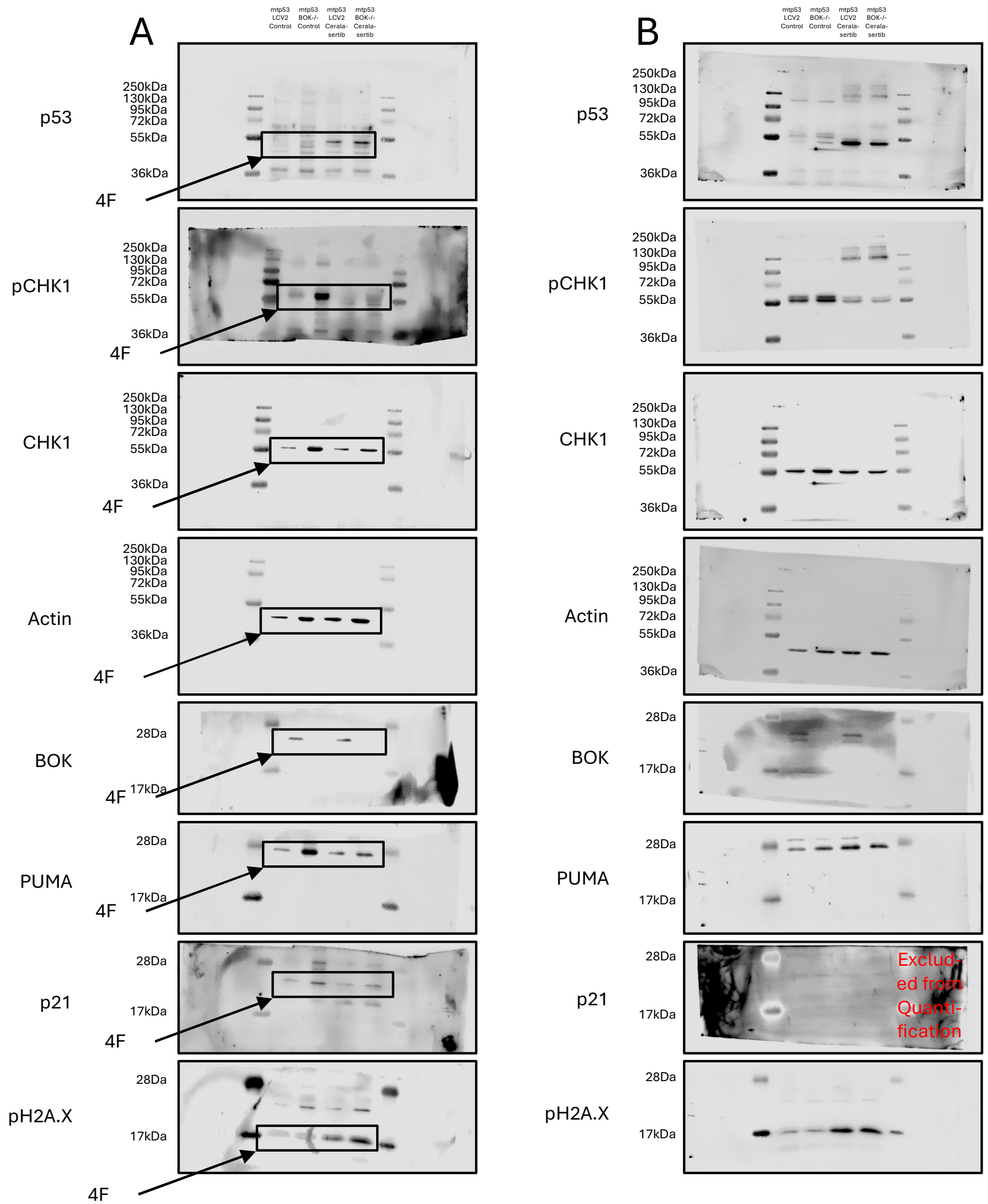

p53[mt] NSCLC Western Blots. A: Whole blot signals from western blot Experiment 310. This western blot was used as a representative blot in Figure 4F. B: Whole blot signals from western blot Experiment 314. All conditions are NSCLC cells. The PVDF membrane was cut at approximately 30 kDa.

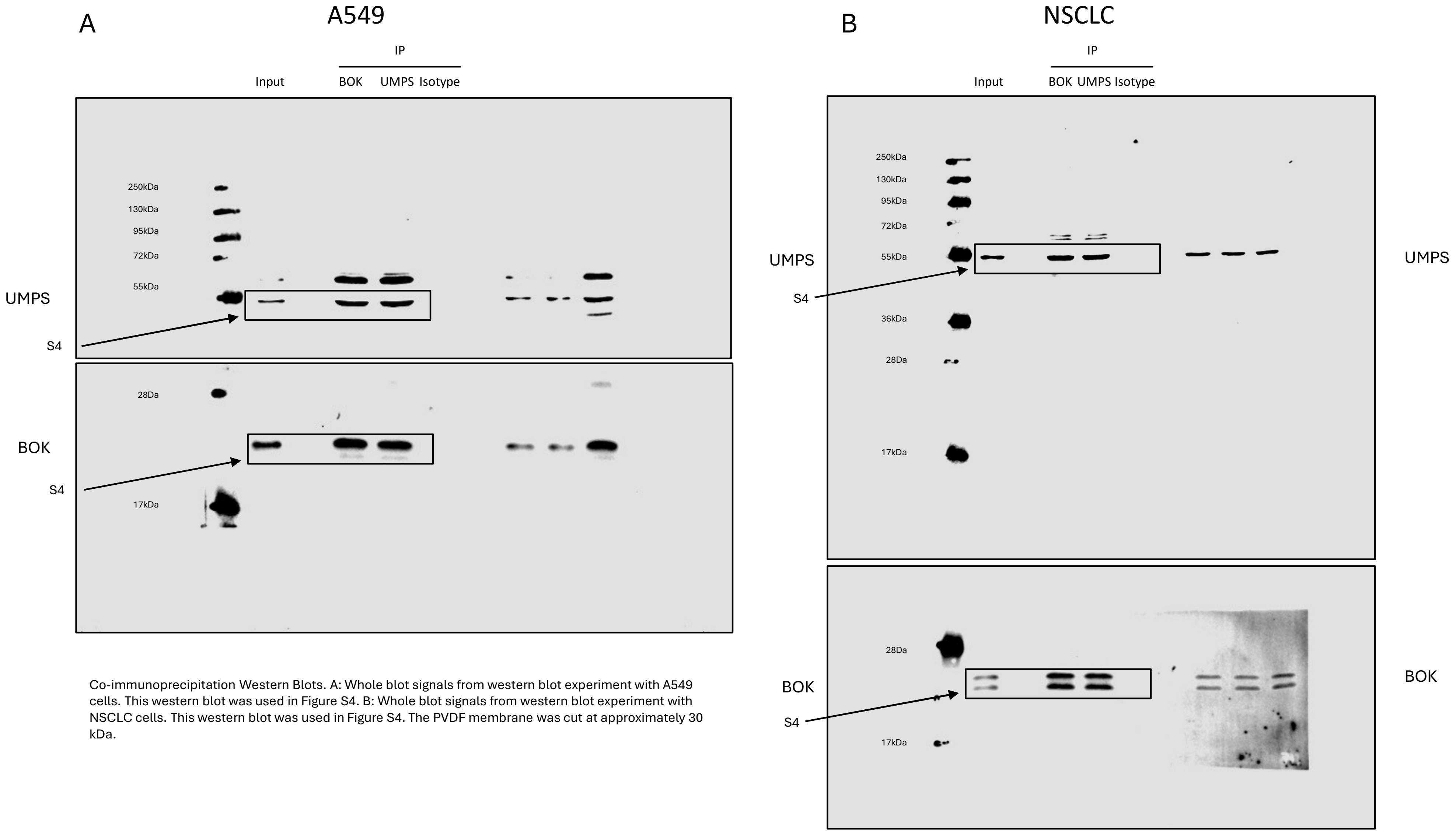

Co-immunoprecipitation Western Blots. A: Whole blot signals from western blot experiment with A549 cells. This western blot was used in Figure S4. B: Whole blot signals from western blot experiment with NSCLC cells. This western blot was used in Figure S4. The PVDF membrane was cut at approximately 30 kDa.
